# Polish healthcare professionals’ perceptions of GLP-1 receptor agonists in cardiometabolic risk reduction: a cross-sectional online survey in Poland

**DOI:** 10.3389/fendo.2026.1906959

**Published:** 2026-08-04

**Authors:** Weronika Faustyna Pucek, Karolina Hoffmann, Wiesław Bryl, Krzysztof Kus, Anna Paczkowska

**Affiliations:** 1 Department of Pharmacoeconomics and Social Pharmacy, Poznan University of Medical Sciences, Poznań, Poland; 2 Department and Clinic of Internal Diseases and Metabolic Disorders, Poznan University of Medical Sciences, Poznań, Poland

**Keywords:** cardiometabolic prevention, cardiovascular risk, GLP-1 receptor agonists, healthcare professionals, obesity

## Abstract

**Background:**

Cardiovascular diseases and metabolic disorders, including obesity and type 2 diabetes, remain leading global health challenges. Despite major advances in pharmacotherapy, the implementation of effective cardiometabolic prevention strategies remains suboptimal. Incretin-based therapies, particularly glucagon-like peptide-1 receptor agonists (GLP-1 RAs), have substantially transformed the management of metabolic diseases by providing benefits beyond glycaemic control, including sustained weight loss, improvements in metabolic parameters, and cardiovascular risk reduction.

**Objectives:**

To evaluate Polish healthcare professionals’ perceptions of GLP-1 receptor agonists in cardiometabolic risk management, with particular emphasis on perceived effectiveness, safety, selected psychological and behavioural effects, and reimbursement.

**Design:**

Cross-sectional online survey conducted among healthcare professionals in Poland between May and August 2025.

**Methods:**

A structured, anonymous online questionnaire was disseminated through professional, institutional, and academic communication channels in Poland. The questionnaire assessed self-reported perceptions, beliefs, perceived clinical value, perceived safety, and implementation-related views regarding GLP-1 receptor agonists in cardiometabolic risk management. The final analytical sample included 1,100 healthcare professionals representing physicians, nurses, pharmacists, dietitians, and physiotherapists. Categorical responses were compared between professional groups using the chi-square test or Fisher’s exact test, as appropriate. Multivariable logistic regression was used to evaluate associations between respondent characteristics and selected perceptions; adjusted odds ratios (ORs) with 95% confidence intervals (CIs) and p-values were reported.

**Results:**

The final analytical sample included 1,100 healthcare professionals: physicians (n = 164), pharmacists (n = 133), nurses (n = 160), physiotherapists (n = 361), and dietitians (n = 282). GLP-1 receptor agonists were most frequently rated as “very effective” for weight loss by physiotherapists (87.3%) and dietitians (84.8%), followed by nurses (59.4%) and physicians (52.4%). Perceived safety was high across all groups, with GLP-1 receptor agonists considered safe by 94.0% of dietitians, 93.9% of physiotherapists, 87.2% of physicians, 85.0% of pharmacists, and 69.4% of nurses. Support for reimbursement was also high, ranging from 75.0% among nurses to 97.2% among dietitians. In multivariable analyses among physicians, employment in a diabetology or endocrinology setting was strongly associated with perceiving GLP-1 receptor agonists as very effective for weight reduction (adjusted OR 11.80, 95% CI 3.88–35.84; p < 0.001), as well as with perceived stress reduction (adjusted OR 2.71, 95% CI 1.05–7.02; p = 0.039), anxiety reduction (adjusted OR 7.00, 95% CI 2.01–24.42; p = 0.002), and improvement in depressive symptoms (adjusted OR 5.81, 95% CI 1.68–20.07; p = 0.005).

**Conclusions:**

Among participating healthcare professionals in Poland, GLP-1 receptor agonists were generally perceived as effective and safe options for cardiometabolic risk management. Perceptions differed across professional groups, particularly regarding weight reduction, safety, psychological and behavioural effects, and reimbursement. Because educational gaps, implementation barriers, real-world adoption, and interdisciplinary care models were not directly assessed as primary outcomes, these areas should be addressed in future studies using dedicated instruments.

## Introduction

Cardiovascular diseases (CVDs), obesity, and type 2 diabetes mellitus (T2DM) remain major and interrelated public health challenges. Their coexistence substantially increases cardiometabolic risk and requires prevention strategies that address body weight (BW), glycaemic control, blood pressure (BP), lipid profile, and long-term cardiovascular (CV) outcomes (1, 2).

Glucagon-like peptide-1 receptor agonists (GLP-1 RAs) and related incretin-based therapies have become important therapeutic options in this context. Their clinical relevance is supported by evidence showing improvements in glycaemic control, weight reduction (WR), and selected cardiometabolic outcomes, including CV benefit in high-risk populations (3–8). GLP-1 RAs enhance glucose-dependent insulin secretion, suppress glucagon secretion, delay gastric emptying, reduce appetite, and may indirectly improve several cardiometabolic risk factors through WR and metabolic regulation (3–6).

The cardiovascular evidence base has expanded beyond the original cardiovascular outcome trials. Recent meta-analytic evidence suggests that baseline age and body mass index may modify the relative reduction in major adverse cardiovascular events, while real-world studies indicate that sustained GLP-1 RA exposure is associated with lower cardiovascular and all-cause mortality and also reveal underuse in several patient groups (9–11). These findings underline the importance of phenotype-specific treatment selection, persistence, adherence, and equitable access when translating trial efficacy into routine clinical effectiveness.

The therapeutic landscape is also moving from injectable peptides towards oral formulations. Oral semaglutide established the feasibility of oral GLP-1 receptor agonism, and orforglipron, a non-peptide small-molecule agonist, has produced clinically meaningful reductions in body weight and HbA1c together with favourable changes in cardiovascular risk biomarkers (12–16). Oral administration may reduce injection-related barriers, but gastrointestinal tolerability, affordability, adherence, drug-specific dosing requirements, and the absence of completed cardiovascular outcome trials for newer agents remain important considerations. Biomarker improvement and weight loss should therefore not be interpreted as evidence of reduced cardiovascular events.

The evidence base is broadening beyond conventional T2DM and obesity populations. Adjunctive GLP-1 RA therapy in T1DM has produced modest reductions in HbA1c and insulin dose but remains investigational and does not replace insulin (17). In HFpEF, frailty, and post-stroke care, current evidence supports phenotype- and comorbidity-driven selection and complementary use of GLP-1 RAs and SGLT2 inhibitors rather than uniform preference for one class (18–21). Multiomics findings suggest inflammatory, metabolic, endothelial, mitochondrial, and remodelling pathways that require clinical confirmation (19). Long-term cardiovascular analyses support an overall favourable efficacy-safety balance in high-risk populations, while uncommon pulmonary and peri-procedural safety signals require continued pharmacovigilance (22, 23). These rapidly evolving areas provide relevant context for interpreting professional perceptions but were not directly assessed by the questionnaire.

However, the translation of GLP-1 RA evidence into routine clinical practice depends not only on trial efficacy, but also on healthcare professionals (HPs)’ familiarity, confidence, prescribing habits, counselling practices, and awareness of implementation barriers. Previous surveys have shown that these factors may differ substantially across professional groups and healthcare systems. In the United Kingdom, Matza et al. surveyed 670 physicians treating patients with T2DM and found broad use of GLP-1 RAs, but also reported that almost one quarter of general practitioners felt they did not have sufficient knowledge to prescribe these therapies (24). An international ACNAP (Association of Cardiovascular Nursing & Allied Professions) survey of CV HPs highlighted practical barriers to injectable therapies with CV benefit, including resource constraints, lack of knowledge among colleagues, paperwork burden, limited patient knowledge, fear of injections, and administration-related concerns (25).

Additional evidence indicates that implementation barriers extend beyond patient acceptance of injectable therapy. Surveys and practice-based studies in the United States identified cost, prior authorization requirements, limited prescribing comfort, and insufficient experience as important barriers to prescribing GLP-1 RAs and other glucose-lowering therapies with cardiometabolic benefit (26, 27). More recent survey data among HPs prescribing anti-obesity medications (AOMs) in the United States showed marked differences in familiarity with GLP-1, glucose-dependent insulinotropic polypeptide (GIP), and glucagon RAs across specialties, suggesting persistent educational needs even in settings where these drugs are increasingly used (28). Similarly, a national survey of Chinese physicians demonstrated knowledge gaps and variation in prescribing patterns for GLP-1 RAs and sodium-glucose cotransporter 2 (SGLT2) inhibitors, while a Croatian primary-care survey identified experience, practice setting, and perceived barriers as relevant determinants of prescribing antidiabetic medications with CV benefits (29, 30).

Taken together, the available literature suggests that knowledge, confidence, prescribing experience, access barriers, and patient-counselling issues remain important obstacles to optimal implementation of GLP-1 RA therapy. Nevertheless, most previous surveys have focused mainly on physicians, selected specialties, cardiometabolic prescribing in T2DM, or injectable therapies more broadly. Fewer studies have evaluated multidisciplinary perceptions including non-prescribing professionals such as nurses, dietitians, physiotherapists, and pharmacists, who may play an important role in patient education, lifestyle support, adherence, monitoring, and long-term cardiometabolic care. Data from Central and Eastern European healthcare settings are also limited, and Polish multidisciplinary evidence is scarce.

Therefore, the present study was designed to address this gap by evaluating Polish HPs’ perceptions of GLP-1 RAs and dual GIP/GLP-1 RAs in cardiometabolic risk management. Its unique contribution lies in the inclusion of several professional groups involved in obesity, T2DM, and CV prevention; the assessment of perceived efficacy, safety, behavioural and psychological effects, reimbursement, and implementation barriers; and the focus on interdisciplinary educational and implementation needs in the Polish healthcare context.

## Materials and methods

### Study design and reporting

This study was designed as a cross-sectional observational online survey conducted among healthcare professionals in Poland between May and August 2025. The study is reported in accordance with the Strengthening the Reporting of Observational Studies in Epidemiology (STROBE) statement for cross-sectional studies. Because the questionnaire was administered online, key elements recommended by the Checklist for Reporting Results of Internet E-Surveys (CHERRIES) were additionally considered, particularly survey access, denominator definition, handling of incomplete responses, duplicate-response prevention, and missing-data reporting.

### Recruitment and sampling

#### Inclusion criteria

Participants were eligible if they met all of the following criteria:

- licensed HPs (physicians, pharmacists, nurses, physiotherapists, or dietitians),
- active clinical or professional practice at the time of the survey,
- professional involvement in the care of patients with T2DM, obesity, or CVD,
- familiarity with, or professional exposure to, incretin-based therapies,
- voluntary consent to participate in the study.

#### Exclusion criteria

- non-healthcare professions,
- incomplete or inconsistently completed questionnaires,
- withdrawal of consent or failure to submit the final survey.

### Recruitment and geographical scope

Participants were recruited using a non-probability convenience sampling strategy through professional, institutional, and academic communication channels in Poland. The survey link was disseminated through healthcare professional networks, institutional and university communication channels, clinical and academic contacts, and professional online communication groups. The survey was available to eligible healthcare professionals across Poland and was not restricted to a single city, institution, or region. This geographical scope of dissemination does not indicate probability-based sampling or geographical representativeness of the final sample. No regional sampling frame, voivodeship-level quotas, or stratified recruitment targets were used. Therefore, the results should be interpreted as reflecting the perceptions of the participating healthcare professionals rather than as representative estimates for all healthcare professionals in Poland.

### Online survey administration and CHERRIES elements

The questionnaire was administered electronically as an anonymous, self-completed online survey. Before entering the questionnaire, potential participants received information about the study aim, voluntary nature of participation, anonymous data collection, eligibility criteria and estimated scope of the survey. Submission of the completed questionnaire was treated as informed consent to participate. No financial or material incentives were offered. The survey was open in the sense that the link could be accessed by individuals who received it through professional dissemination channels; however, eligibility was restricted to HPs meeting the predefined inclusion criteria.

### Questionnaire development, content and response options

The study used a study-specific questionnaire developed by the authors for the purposes of this survey. No previously validated questionnaire directly addressing multidisciplinary healthcare professionals’ perceptions of GLP-1 RAs in cardiometabolic risk management in Poland was available; therefore, a dedicated instrument was prepared.

The questionnaire was developed on the basis of current clinical and pharmacological knowledge regarding GLP-1 RAs, the study objectives, and the professional experience of the research team in endocrinology, pharmacoeconomics, social pharmacy, and cardiometabolic prevention. The draft questionnaire was reviewed by members of the multidisciplinary research team to assess content relevance, clarity, and face validity. Items were revised to ensure that they were understandable to different groups of HPs, including physicians, pharmacists, nurses, physiotherapists, and dietitians.

The questionnaire included the following thematic sections: sociodemographic and professional characteristics; professional experience and clinical exposure to patients with obesity, T2DM, or cardiometabolic risk; perceived effectiveness of GLP-1 RAs in WR and metabolic-risk management; perceived effects on selected biochemical, psychological, and behavioural outcomes; perceived safety and adverse events (AEs); views on access, reimbursement, and implementation barriers; and perceived role of interdisciplinary care.

Response options varied according to item type. Demographic and professional variables were collected using categorical or numerical response fields. Perception-based items used closed categorical response options, including “yes”, “no”, and “uncertain”, or ordered response categories such as “very effective”, “effective”, and “ineffective”, depending on the question. Items concerning perceived AEs allowed respondents to indicate predefined AE categories.

The questionnaire was not designed as an objective knowledge test. It did not include a formal scoring system for factual knowledge, and no total knowledge score was calculated. Respondents were therefore not classified as having “good”, “moderate”, or “poor” knowledge. The analysed outcomes represent self-reported perceptions, beliefs, confidence, and professional views regarding GLP-1 RAs. For this reason, all results are interpreted as perception-based and not as objective measures of clinical knowledge or competence.

For statistical analysis, selected questionnaire responses were analysed as categorical variables. Where applicable, binary outcomes were created for regression modelling, for example perceived “very high effectiveness” for weight loss versus other responses, or perceived presence versus absence/uncertainty of selected psychological or behavioural effects. The complete questionnaire is provided as **Supplementary Material 4**.

### Questionnaire validation and psychometric characteristics

The questionnaire was reviewed internally by members of the multidisciplinary research team for content relevance, clarity, comprehensibility, and face validity, and individual items were revised accordingly. No quantitative content-validity index was calculated. No formal pilot testing involving members of the target population was conducted. Because the instrument was developed specifically for this exploratory cross-sectional survey and was not intended to generate a total score or thematic-section-specific composite scores, no construct-validation procedure, including exploratory factor analysis, was performed. The heterogeneous categorical items were analysed individually; therefore, internal-consistency coefficients such as Cronbach’s alpha were not calculated because no unidimensional multi-item scale had been defined. These limitations are acknowledged in the Discussion.

### Participant flow and denominator reporting

A total of 1,100 complete and eligible questionnaires were retained in the final analytical dataset. Analytics on the number of unique individuals reached, landing-page views and questionnaire starts were not recorded in a form that allowed reliable calculation of view, participation or completion rates. Therefore, these rates are reported as not calculable. Incomplete, ineligible, duplicate or internally inconsistent records were not retained in the analytical dataset. The final analysis was based on 1,100 eligible HPs from Poland.

### Sample-size rationale

No formal *a priori* sample-size calculation was performed. Recruitment was conducted during the predefined survey period from May to August 2025, and the achieved sample size was determined by the number of complete and eligible questionnaires obtained during that period. To contextualise the precision of the final sample, a proportion-based calculation was performed using a 95% confidence level, an expected proportion of 50%, and an absolute precision of ±3 percentage points. Under these assumptions, the required sample size was approximately 1,068 respondents. The final analytical sample of 1,100 exceeded this value and corresponded to a maximum nominal margin of error of approximately ±2.95 percentage points under simple random-sampling assumptions. Because the study used non-probability convenience sampling, this calculation reflects nominal statistical precision and should not be interpreted as evidence of population representativeness.

### Duplicate and data-quality control

To protect respondent anonymity, direct personal identifiers were not collected. IP addresses, cookies, login-based identifiers, names, telephone numbers, e-mail addresses, and workplace identifiers were not included in the analytical dataset. Therefore, duplicate screening was based on response-pattern assessment rather than direct respondent identification.

Data-quality criteria were defined before the final statistical analysis and were applied consistently to the exported dataset. Screening was conducted in two steps. First, records were flagged using predefined logical and plausibility rules. Second, flagged records were manually reviewed before any exclusion decision was made. No record was excluded solely because of a single unusual but plausible answer.

An internally inconsistent response pattern was defined as one or more of the following: reporting a professional group outside the predefined eligible categories while simultaneously completing profession-specific sections intended for eligible HPs; reporting no active professional practice despite meeting other professional-exposure criteria; providing mutually incompatible professional responses, such as selecting a professional role and a work setting that were logically incompatible with the eligibility criteria; reporting years (yr.) of professional practice greater than the respondent’s age minus the minimum plausible age at professional qualification; or providing contradictory answers to related clinical-exposure items. Response patterns were also considered internally inconsistent when a respondent gave the same response option across all substantive items in a way that conflicted with filter or profession-specific questions and suggested non-differentiated responding.

Implausible demographic values were defined as age values outside the plausible range for licensed HPs in Poland or professional-experience values incompatible with the reported age. In the present study, age below 22 yr. or above 80 yr. was considered implausible for the target population. Years of professional practice below 0 or above 60 yr. were considered implausible. In addition, years of professional practice exceeding age minus 18 yr. were flagged as logically implausible and manually reviewed.

Suspected duplicate records were defined as records with identical or near-identical combinations of core demographic and professional characteristics, including professional group, sex, age, years of practice, specialist work setting, and identical responses across the main perception-based questionnaire items. Because no direct identifiers were available, records were not considered duplicates on demographic similarity alone. A record was excluded as a suspected duplicate only when demographic/professional characteristics and substantive response patterns were identical or near-identical and there was no reasonable indication that they represented independent respondents.

Ambiguous cases were resolved conservatively. Records were excluded only when the predefined criteria provided clear evidence of ineligibility, internal inconsistency, implausible values, or suspected duplication. If the review did not allow a confident exclusion decision, the record was retained. The final analytical dataset included only complete, eligible, and internally consistent questionnaires.

### Missing data and statistical handling

Before analysis, the dataset was screened for eligibility, completeness of key sections, implausible demographic values, internally inconsistent answer patterns, and duplicate-like records. The final analytical dataset included 1,100 complete and eligible questionnaires. Analyses were conducted using available valid observations for each variable. Multivariable logistic regression models were estimated using complete-case analysis for the variables included in each model. The number of valid observations included in descriptive analyses and the number of observations included in each multivariable model are reported in the corresponding tables.

### Normality and collinearity diagnostics

The distribution of continuous variables was assessed using visual inspection of histograms and the Shapiro–Wilk test. Continuous variables were summarised as means with standard deviations or medians with interquartile ranges, as appropriate. Before multivariable modelling, the relationship between age and years of professional practice was assessed using Pearson correlation coefficients, variance inflation factors, and tolerance statistics. In the physician models, both variables were retained because the variance inflation factors remained below the decision threshold applied during final model development, although collinearity was substantial and the corresponding coefficients were interpreted cautiously. In the non-physician models, age was omitted when the initial variance inflation factors exceeded 10 or tolerance values were below 0.10, and years of professional practice was retained because of its greater professional interpretability.

### Ethics approval and consent to participate

The study was reviewed and approved by the Bioethics Committee of Poznan University of Medical Sciences, Poznań, Poland (approval no. KB-301/25; 14 May 2025). The study was conducted in accordance with the Declaration of Helsinki. Participation was voluntary and anonymous. Before accessing the questionnaire, participants received information about the study objectives, scope, non-interventional nature, anonymity of data collection, and their right not to participate. Submission of the completed questionnaire was considered informed consent to participate. No personal identifiers or patient-level data were collected.

### Statistical analysis

Statistical analyses were performed using IBM SPSS Statistics, version 27.0. Categorical variables were summarized as frequencies and percentages. Continuous variables were summarized as means with standard deviations (SDs) or medians (Mdns) with interquartile ranges, depending on distribution.

Between-group differences in categorical variables were analysed using the chi-square test or Fisher’s exact test, as appropriate. Cramer’s V was reported as an effect-size measure for selected between-group comparisons. Multivariable logistic regression analyses were performed for selected perception-based outcomes. Variables entered into the final models were selected on the basis of clinical and methodological relevance; univariable statistical significance was not used as the sole inclusion criterion. Age, sex, years of professional practice, and, in physician models, employment in a diabetology- or endocrinology-related setting were considered potential explanatory or adjustment variables, as applicable. All covariates included in each final model are reported irrespective of statistical significance. No interaction terms were included in the final models because the analyses were exploratory and some outcome categories or professional subgroups contained limited numbers of events. Continuous predictors were entered per one-year increase and therefore assumed linearity on the logit scale. Model fit was evaluated using the −2 log likelihood, Nagelkerke R², the Hosmer–Lemeshow goodness-of-fit test, and the area under the receiver operating characteristic curve. Collinearity was assessed using Pearson correlations, variance inflation factors, and tolerance values. Formal analyses of interaction effects and influential observations were not undertaken. Adjusted odds ratios, 95% confidence intervals, exact p-values, model sample sizes, outcome-event counts, model-fit statistics, and collinearity diagnostics are reported in **Table 1** and its continuation. All tests were two-sided, and p < 0.05 was considered statistically significant. Because the modelling analyses were exploratory and involved multiple coefficient tests, results close to the conventional significance threshold were interpreted cautiously.

**Table 1:** Complete multivariable logistic regression models for selected perceptions of GLP-1 RAs.

Model	Group	Outcome	n	Events n (%)	Predictor	Reference/unit	aOR	95% CI	p	VIF	Tolerance
M1	Physicians	Very high effectiveness for weight loss	164	86 (52.4)	Age (per 1-year increase)	per 1-year increase	0.96	0.87–1.07	0.490	8.21	0.122
M1	Physicians	Very high effectiveness for weight loss	164	86 (52.4)	Female sex (vs male)	male	0.50	0.25–0.99	0.046	1.01	0.990
M1	Physicians	Very high effectiveness for weight loss	164	86 (52.4)	Years in practice (per 1-year increase)	per 1-year increase	1.03	0.93–1.14	0.537	8.20	0.122
M1	Physicians	Very high effectiveness for weight loss	164	86 (52.4)	Diabetology/endocrinology setting (yes vs no)	no	11.80	3.88–35.84	<0.001	1.00	0.999
M2	Physicians	Perceived reduction of stress	164	113 (68.9)	Age (per 1-year increase)	per 1-year increase	1.01	0.91–1.13	0.801	8.21	0.122
M2	Physicians	Perceived reduction of stress	164	113 (68.9)	Female sex (vs male)	male	0.83	0.42–1.64	0.597	1.01	0.990
M2	Physicians	Perceived reduction of stress	164	113 (68.9)	Years in practice (per 1-year increase)	per 1-year increase	0.99	0.89–1.09	0.773	8.20	0.122
M2	Physicians	Perceived reduction of stress	164	113 (68.9)	Diabetology/endocrinology setting (yes vs no)	no	2.71	1.05–7.02	0.039	1.00	0.999
M3	Physicians	Perceived reduction of anxiety	164	113 (68.9)	Age (per 1-year increase)	per 1-year increase	0.81	0.66–0.99	0.039	8.21	0.122
M3	Physicians	Perceived reduction of anxiety	164	113 (68.9)	Female sex (vs male)	male	1.64	0.81–3.31	0.171	1.01	0.990
M3	Physicians	Perceived reduction of anxiety	164	113 (68.9)	Years in practice (per 1-year increase)	per 1-year increase	1.24	1.01–1.51	0.040	8.20	0.122
M3	Physicians	Perceived reduction of anxiety	164	113 (68.9)	Diabetology/endocrinology setting (yes vs no)	no	7.00	2.01–24.42	0.002	1.00	0.999
M4	Physicians	Perceived reduction of depressive symptoms	164	117 (71.3)	Age (per 1-year increase)	per 1-year increase	0.99	0.88–1.11	0.850	8.21	0.122
M4	Physicians	Perceived reduction of depressive symptoms	164	117 (71.3)	Female sex (vs male)	male	0.80	0.39–1.61	0.526	1.01	0.990
M4	Physicians	Perceived reduction of depressive symptoms	164	117 (71.3)	Years in practice (per 1-year increase)	per 1-year increase	1.03	0.91–1.15	0.668	8.20	0.122
M4	Physicians	Perceived reduction of depressive symptoms	164	117 (71.3)	Diabetology/endocrinology setting (yes vs no)	no	5.81	1.68–20.07	0.005	1.00	0.999
M5	Nurses	Perceived reduction of stress	160	65 (40.6)	Female sex (vs male)	male	0.54	0.22–1.30	0.170	1.03	0.973
M5	Nurses	Perceived reduction of stress	160	65 (40.6)	Years in practice (per 1-year increase)	per 1-year increase	0.95	0.92–0.98	0.002	1.03	0.973
M6	Physiotherapists	Perceived reduction of stress	361	257 (71.2)	Female sex (vs male)	male	1.09	0.69–1.72	0.711	1.00	0.999
M6	Physiotherapists	Perceived reduction of stress	361	257 (71.2)	Years in practice (per 1-year increase)	per 1-year increase	0.99	0.95–1.04	0.779	1.00	0.999
M7	Dietitians	Perceived reduction of depressive symptoms	282	186 (66.0)	Female sex (vs male)	male	4.69	0.88–24.85	0.069	1.00	0.998
M7	Dietitians	Perceived reduction of depressive symptoms	282	186 (66.0)	Years in practice (per 1-year increase)	per 1-year increase	1.08	1.02–1.14	0.010	1.00	0.998
M8	Pharmacists	Very high effectiveness for weight loss based on patients’ feedback	133	85 (63.9)	Female sex (vs male)	male	0.69	0.32–1.48	0.338	1.02	0.979
M8	Pharmacists	Very high effectiveness for weight loss based on patients’ feedback	133	85 (63.9)	Years in practice (per 1-year increase)	per 1-year increase	0.93	0.89–0.98	0.011	1.02	0.979
Model-level coding, variables entered, and fit statistics.											
Model	Group	Outcome	Outcome coding		Variables entered	−2 log likelihood	Nagelkerke R²	Hosmer–Lemeshow χ² (df)		Hosmer–Lemeshow p	AUC
M1	Physicians	Perceived very high effectiveness for weight loss	1 = very effective; 0 = effective or ineffective		Age (per 1-year increase); female sex (reference: male); years in practice (per 1-year increase); specialist setting (reference: no)	195.129	0.235	11.181 (8)		0.192	0.732
M2	Physicians	Perceived reduction of stress	1 = yes; 0 = no or uncertain		Age (per 1-year increase); female sex (reference: male); years in practice (per 1-year increase); specialist setting (reference: no)	198.037	0.045	5.179 (8)		0.738	0.616
M3	Physicians	Perceived reduction of anxiety	1 = yes; 0 = no or uncertain		Age (per 1-year increase); female sex (reference: male); years in practice (per 1-year increase); specialist setting (reference: no)	181.920	0.172	4.852 (8)		0.773	0.713
M4	Physicians	Perceived reduction of depressive symptoms	1 = yes; 0 = no or uncertain		Age (per 1-year increase); female sex (reference: male); years in practice (per 1-year increase); specialist setting (reference: no)	184.432	0.102	5.894 (8)		0.659	0.627
M5	Nurses	Perceived reduction of stress	1 = yes; 0 = no or uncertain		Female sex (reference: male); years in practice (per 1-year increase)	201.392	0.119	7.254 (8)		0.509	0.664
M6	Physiotherapists	Perceived reduction of stress	1 = yes; 0 = no or uncertain		Female sex (reference: male); years in practice (per 1-year increase)	433.290	0.001	6.001 (8)		0.647	0.509
M7	Dietitians	Perceived reduction of depressive symptoms	1 = yes; 0 = no or uncertain		Female sex (reference: male); years in practice (per 1-year increase)	350.163	0.055	12.485 (8)		0.131	0.623
M8	Pharmacists	Perceived very high effectiveness for weight loss based on patients’ feedback	1 = very effective; 0 = effective or ineffective		Female sex (reference: male); years in practice (per 1-year increase)	166.545	0.074	2.204 (8)		0.974	0.629

Outcome coding: for effectiveness models, 1 = very effective and 0 = effective/ineffective; for yes/no/uncertain items, 1 = yes and 0 = no/uncertain. ORs are adjusted odds ratios. p-values are exact to three decimals, except p < 0.001. Collinearity rule: age and years in practice were initially assessed together; where VIF was ≥10 in non-physician groups, age was omitted and years in practice was retained due to greater professional relevance.

aOR, adjusted odds ratio; AUC, area under the receiver operating characteristic curve; CI, confidence interval; df, degrees of freedom; HL, Hosmer–Lemeshow test; LL, log likelihood; VIF, variance inflation factor.

Outcome coding defines the modelled event as 1. Specialist setting denotes employment in a diabetology- or endocrinology-related setting. For models M5–M8, age was omitted from the final models because of substantial collinearity with years of professional practice.

## Results

### Characteristics of the study group

The survey was distributed through professional, institutional and academic communication channels in Poland. Because the survey link was disseminated through open and network-based channels, the exact number of individuals who received or opened the survey could not be determined. A total of 1,100 complete and internally consistent questionnaires were included in the final analytical sample. Incomplete, duplicate or inconsistent questionnaires were excluded before analysis. A participant-flow and data-quality screening summary is provided as **Supplementary Material 3**. All records retained in the final analytical dataset met the predefined eligibility and data-quality criteria. Records flagged during data-quality screening were reviewed manually. Exclusions were made only when there was clear evidence of ineligibility, internal inconsistency, implausible demographic or professional values, or suspected duplication. Ambiguous records were retained.

A total of 1,100 HPs participated in the survey, including physicians (n=164), pharmacists (n=133), nurses (n=160), physiotherapists (n=361), and dietitians (n=282). Women constituted the majority in all groups except physicians and pharmacists, with the highest proportion observed among dietitians (97.5%).

The mean age ranged from 31.2 ± 5.0 years among dietitians to 37.0 ± 8.8 years among physicians. Nurses exhibited the greatest age variability (SD = 11.1), consistent with their wider age range (23–70 years).

Years of professional experience mirrored age distributions. Nurses reported the longest practice duration (13.1 ± 11.6 yr.), whereas physiotherapists and dietitians reported comparatively shorter experience (9.0 ± 5.1 and 8.6 ± 4.9 yr., respectively).

Together, these data describe a heterogeneous multidisciplinary sample of healthcare professionals involved in obesity- and T2DM-related care (**Table 2**).

**Table 2:** Baseline characteristics of the study group.

Characteristic	Physicians	Pharmacists	Nurses	Physiotherapists	Dietitians
Female, n (%)	85 (51.83)	79 (59.40)	134 (83.75)	200 (55.40)	275 (97.52)
Male, n (%)	79 (48.17)	54 (40.60)	26 (16.25)	161 (44.60)	7 (2.48)
Age, years, mean ± SD	37.0 ± 8.8	35.1 ± 7.8	36.5 ± 11.1	31.2 ± 5.2	31.2 ± 5.0
Age range	26–70	26–70	23–70	23–55	22–55
Years in practice, mean ± SD	11.2 ± 9.1	8.9 ± 7.4	13.1 ± 11.6	9.0 ± 5.1	8.6 ± 4.9
Years in practice range	1–45	1–40	1–48	1–27	1–32

### Comparative analysis of the effectiveness of GLP-1 RAs in WR and their impact on blood biochemical parameters, including lipid profile and carbohydrate metabolism

Across professional groups, respondents differed in their perception of GLP-1 RA effectiveness for weight loss. The proportion rating GLP-1 RAs as “very effective” was highest among physiotherapists (87.3%) and dietitians (84.8%), followed by nurses (59.4%) and physicians (52.4%). The distribution of responses differed significantly between professional groups (chi-square test, p < 0.001; Cramer’s V = 0.25), indicating a moderate association between professional group and perceived weight-loss effectiveness (**Table 3**).

**Table 3:** Perceived clinical effectiveness of GLP-1 RAs in cardiometabolic risk management (between-professional-group comparison for perceived weight-loss effectiveness (chi-square test, p < 0.001; Cramer’s V = 0.25).

Outcome	Physicians	Nurses	Physiotherapists	Dietitians
Very effective for weight loss (%)	52.44	59.38	87.26	84.75
Effective (%)	46.95	37.50	12.74	14.89
Ineffective (%)	0.61	3.13	0	0.35
Reduces metabolic syndrome risk (%)	98.78	—	98.89	99.29
Reduces HbA1c (%)	89.02	70.00	—	—
Reduces LDL (%)	77.44	51.88	—	—
Reduces HDL (%)	73.17	46.25	—	—
Reduces non-HDL (%)	60.37	41.88	—	—
Reduces triglycerides (%)	59.76	43.75	—	—
Reduces total cholesterol (%)	63.41	45.63	—	—

Data are shown only for professional groups to whom the corresponding questionnaire items were administered and applicable according to the questionnaire pathway. Therefore, professional groups not shown in this table did not receive the corresponding items or were not included in that item-specific analysis.

Almost all respondents in the professional groups to whom this item was applicable perceived GLP-1 RAs as contributing to a reduction in metabolic-syndrome risk, with endorsement rates exceeding 98% among physicians, physiotherapists, and dietitians. Physicians and nurses also frequently perceived GLP-1 RAs as associated with improvement in glycaemic control, with 89.0% and 70.0%, respectively, reporting perceived glycated haemoglobin (HbA1c) reduction (**Table 3**).

Perceived lipid-profile benefits were reported more frequently by physicians than by nurses. Physicians more often perceived reductions in low-density lipoprotein cholesterol (LDL-C, 77.4%), high-density lipoprotein cholesterol (HDL-C, 73.2%), non-high-density lipoprotein cholesterol (non-HDL-C, 60.4%), triglycerides (TG, 59.8%), and total cholesterol (TC, 63.4%), whereas nurses reported these perceived effects at lower frequencies. These results reflect respondents’ professional perceptions rather than objectively measured biochemical outcomes (**Table 3**).

### HPs’ perceptions of the possible effects of GLP-1 RAs on stress, depressive symptoms, and anxiety

HPs differed in their perceptions of possible psychological effects of GLP-1 RA therapy. Perceived stress reduction was reported by 68.9% of physicians, 71.2% of physiotherapists, and 67.7% of dietitians, compared with 40.6% of nurses. The distribution of responses differed significantly across professional groups (chi-square test, p < 0.001; Cramer’s V = 0.22). These findings describe perceived effects only and should not be interpreted as observed changes in patient stress levels (**Table 4**).

**Table 4:** Perceived effects of GLP-1 RAs on stress, anxiety and depressive symptoms (between-professional-group comparisons: perceived stress reduction, p < 0.001, Cramer’s V = 0.22; perceived anxiety reduction, p < 0.001, Cramer’s V = 0.27; perceived improvement in depressive symptoms, p < 0.001, Cramer’s V = 0.18).

Outcome	Physicians	Nurses	Physiotherapists	Dietitians
Reduces stress (%)	68.90	40.63	71.19	67.73
No (%)	21.95	40.00	8.03	15.60
Uncertain (%)	9.15	19.38	20.78	16.67
Reduces anxiety (%)	68.90	57.50	90.86	91.49
No (%)	14.02	25.63	1.11	5.32
Uncertain (%)	17.07	16.88	8.03	3.19
Reduces depression (%)	71.34	53.75	73.68	65.96
No (%)	12.20	27.50	4.16	13.48
Uncertain (%)	16.46	18.75	22.16	20.57

Perceived anxiety reduction was reported most frequently by physiotherapists (90.9%) and dietitians (91.5%), whereas physicians and nurses reported this perception less frequently (68.9% and 57.5%, respectively). Formal comparison showed significant between-group differences (chi-square test, p < 0.001; Cramer’s V = 0.27) (**Table 4**).

Perceived improvement in depressive symptoms was reported by 71.3% of physicians, 73.7% of physiotherapists, 66.0% of dietitians, and 53.8% of nurses. The distribution of responses differed significantly between professional groups (chi-square test, p < 0.001; Cramer’s V = 0.18) (**Table 4**).

These findings indicate that some respondents, especially those working closely with behavioural or lifestyle interventions, perceived GLP-1 RAs as potentially relevant to mental-health-related domains. However, the survey did not measure patient-level psychological outcomes, and these results should therefore be interpreted only as HPs’ perceptions (**Table 4**).

### HPs’ perceptions of GLP-1 RAs in relation to alcohol consumption and cigarette smoking

Respondents also differed in their perceptions of possible behavioural effects of GLP-1 RA therapy. A perceived reduction in cigarette consumption was reported by 64.6% of physicians and 68.1% of dietitians, compared with 36.9% of nurses. The distribution of responses differed significantly across professional groups (chi-square test, p < 0.001; Cramer’s V = 0.25). These results reflect perceived patient-related effects rather than clinically verified changes in smoking behaviour (**Table 5**).

**Table 5:** Perceived effects of GLP-1 receptor agonists on cigarette smoking and alcohol use (between-professional-group comparisons: perceived reduction in cigarette use, p < 0.001, Cramer’s V = 0.25; perceived reduction in alcohol dependence, p < 0.001, Cramer’s V = 0.25).

Outcome	Physicians	Nurses	Dietitians
Reduces cigarette use (%)	64.63	36.88	68.09
No (%)	15.85	42.50	9.93
Uncertain (%)	19.51	20.63	21.99
Reduces alcohol dependence (%)	56.10	32.50	66.67
No (%)	18.90	44.38	11.35
Uncertain (%)	25.00	23.13	21.99

Data are shown only for professional groups to whom the corresponding questionnaire items were administered and applicable according to the questionnaire pathway. Therefore, professional groups not shown in this table did not receive the corresponding items or were not included in that item-specific analysis.

A similar pattern was observed for perceptions related to alcohol use. A perceived reduction in alcohol dependence was reported by 66.7% of dietitians and 56.1% of physicians, compared with 32.5% of nurses. Between-group differences were statistically significant (chi-square test, p < 0.001; Cramer’s V = 0.25) (**Table 5**).

Across these behavioural domains, nurses more frequently selected “uncertain,” suggesting greater hesitation, lower exposure to such patient reports, or less confidence in interpreting addiction-related outcomes in the context of GLP-1 RA therapy.

### Perceived safety and adverse-event profile of GLP-1 RAs

### Perceived AE profile of GLP-1 RAs in clinical practice

A strong overall perception of safety was observed, although responses differed significantly by professional group (chi-square test, p < 0.001; Cramer’s V = 0.22). The highest proportions of respondents who considered GLP-1 RAs safe were observed among dietitians (94.0%) and physiotherapists (93.9%), followed by physicians (87.2%) and pharmacists (85.0%). Nurses expressed more cautious perceptions, with 69.4% rating GLP-1 RAs as safe and 20.6% selecting “uncertain.” (**Table 6**).

**Table 6:** HPs’ perceptions of the safety profile of incretin-based therapies (between-professional-group comparison for perceived safety: chi-square test, p < 0.001; Cramer’s V = 0.22).

Group	Safe (%)	Not safe (%)	Uncertain (%)
Physicians	87.20	0	12.80
Pharmacists	84.96	3.76	11.28
Nurses	69.38	10.00	20.63
Physiotherapists	93.91	0	6.09
Dietitians	93.97	1.06	4.96

The perceived AE profile was dominated by gastrointestinal (GI) symptoms, including nausea, vomiting, and diarrhoea. These were indicated most frequently by nurses (45.9%), followed by physicians (43.8%), pharmacists (42.0%), and dietitians (35.5%). Injection-site reactions, such as redness, soreness, and swelling, were also commonly indicated, particularly by nurses (30.6%), pharmacists (28.3%), and physicians (23.8%), whereas dietitians reported this category less frequently (12.8%) (**Table 7**).

**Table 7:** HPs’ perceptions of the AE profile of incretin-based therapies (between-professional-group comparison for perceived adverse event profile: (chi-square test, p < 0.001; Cramer’s V = 0.22).

AE	Physicians	Nurses	Pharmacists	Dietitians
Gastrointestinal (GI) problems (%) (nausea, vomiting, diarrhoea)	43.75	45.85	42.03	35.46
Redness, soreness, and swelling of the skin at the injection site (%)	23.75	30.57	28.26	12.77
Hypoglycaemia (%)	8.75	8.30	10.14	2.84
Headache (%)	17.50	8.73	15.22	11.35
Cardiac arrhythmias (%)	0	0.44	0	0
Other (%)	6.25	6.11	4.35	37.59

Data are shown only for professional groups to whom the corresponding questionnaire items were administered and applicable according to the questionnaire pathway. Therefore, professional groups not shown in this table did not receive the corresponding items or were not included in that item-specific analysis.

Hypoglycaemia was perceived as relatively uncommon, with proportions ranging from 2.8% among dietitians to 10.1% among pharmacists. Headache was indicated by 17.5% of physicians, 15.2% of pharmacists, 11.4% of dietitians, and 8.7% of nurses. Cardiac arrhythmias were rarely perceived as AEs. Overall, respondents perceived GLP-1 RAs as safe, while GI symptoms and injection-site reactions constituted the most frequently recognised AEs. These findings represent perceived AE patterns rather than prospectively collected clinical safety outcomes (**Tables 6**, **7**).

### Assessment of availability, reimbursement, and recommendations for the use of GLP-1 RAs

There was strong agreement that GLP-1 RAs should be reimbursed, although responses differed significantly across professional groups (chi-square test, p < 0.001; Cramer’s V = 0.20). Endorsement rates exceeded 85% among physicians, pharmacists, dietitians, and physiotherapists, whereas nurses showed lower but still majority support (75.0%) (**Table 8**).

**Table 8:** HPs’ perceptions of access, reimbursement, and recommendations for incretin-based therapies (between-professional-group comparison for reimbursement support: chi-square test, p < 0.001; Cramer’s V = 0.20).

Group	Reimbursement yes (%)	Reimbursement no (%)	Uncertain (%)
Physicians	88.41	5.49	6.10
Pharmacists	86.47	5.26	8.27
Nurses	75.00	7.50	17.50
Physiotherapists	96.40	0.28	3.32
Dietitians	97.16	0	2.84

Respondents overwhelmingly considered GLP-1 RAs a breakthrough therapy, particularly dietitians (96.5%), physiotherapists (93.6%), and physicians (92.1%) (**Table 8**).

Support for wider dissemination of GLP-1 RA therapy was also high: 81.1% of physicians and 85.0% of pharmacists endorsed broader implementation for obesity treatment (**Table 8**).

These results highlight broad cross-professional support for the therapeutic value and broader accessibility of GLP-1 RAs, while also showing statistically significant differences in perceptions between professional groups.

### Multifactorial determinants of the perceived efficacy of GLP-1 RAs and their psychological impact

Outcome events represent the number of respondents coded as 1 for the corresponding binary outcome. Adjusted odds ratios were estimated using multivariable logistic regression. In physician models, age and years of professional practice were both retained because VIF values remained below the decision threshold of 10 applied during final model development, although moderate collinearity was observed. In non-physician models, age was omitted from final models because initial diagnostics indicated substantial collinearity with years of professional practice; years of professional practice was retained due to its greater professional interpretability. Exact p-values are reported to three decimal places, except values below 0.001, which are reported as p < 0.001.

### Multivariable logistic regression analyses

Complete multivariable logistic regression results are presented in **Table 1**. The table reports the analytical sample size, number and percentage of outcome events, outcome and reference-category coding, all variables entered into each model, adjusted ORs (aORs), 95% CIs, exact p-values, model-fit statistics, and collinearity diagnostics.

In the physician group, four multivariable models were estimated, each including age, sex, years of professional practice, and employment in a diabetology- or endocrinology-related setting. For perceived very high effectiveness of GLP-1 RAs for WR, the model included 164 physicians, with 86 outcome events (52.4%). Employment in a diabetology- or endocrinology-related setting was strongly associated with higher odds of perceiving GLP-1 RAs as very effective for WR (adjusted OR 11.80, 95% CI 3.88–35.84; p < 0.001). Female physicians had lower odds of reporting this perception compared with male physicians (adjusted OR 0.50, 95% CI 0.25–0.99; p = 0.046). Age and years of professional practice were not independently associated with this outcome. The model showed acceptable discrimination, with an AUC of 0.732 and Nagelkerke R² of 0.235.

For perceived stress reduction among physicians, the model included 164 observations and 113 outcome events (68.9%). Employment in a diabetology- or endocrinology-related setting was associated with higher odds of perceiving a stress-reducing effect of GLP-1 RAs (adjusted OR 2.71, 95% CI 1.05–7.02; p = 0.039). Age, sex, and years of professional practice were not significantly associated with this outcome. Model discrimination was modest, with an AUC of 0.616 and Nagelkerke R² of 0.045.

For perceived anxiety reduction among physicians, the model included 164 observations and 113 outcome events (68.9%). Employment in a diabetology- or endocrinology-related setting was associated with markedly higher odds of perceiving an anxiety-reducing effect of GLP-1 RAs (adjusted OR 7.00, 95% CI 2.01–24.42; p = 0.002). Increasing age was associated with lower odds of this perception (adjusted OR 0.81 per one-year increase, 95% CI 0.66–0.99; p = 0.039), whereas longer professional practice was associated with higher odds (adjusted OR 1.24 per one-year increase, 95% CI 1.01–1.51; p = 0.040). Female sex was not significantly associated with the outcome. The model showed acceptable discrimination, with an AUC of 0.713 and Nagelkerke R² of 0.172.

For perceived reduction of depressive symptoms among physicians, the model included 164 observations and 117 outcome events (71.3%). Employment in a diabetology- or endocrinology-related setting was independently associated with higher odds of perceiving a beneficial effect on depressive symptoms (adjusted OR 5.81, 95% CI 1.68–20.07; p = 0.005). Age, sex, and years of professional practice were not significantly associated with this outcome. The model had an AUC of 0.627 and Nagelkerke R² of 0.102.

Among nurses, the final model for perceived stress reduction included 160 observations and 65 outcome events (40.6%). Female sex was not significantly associated with this outcome (adjusted OR 0.54, 95% CI 0.22–1.30; p = 0.170). Longer years of professional practice were associated with lower odds of perceiving GLP-1 RA as reducing stress (adjusted OR 0.95 per one-year increase, 95% CI 0.92–0.98; p = 0.002). The model had an AUC of 0.664 and Nagelkerke R² of 0.119.

Among physiotherapists, the model for perceived stress reduction included 361 observations and 257 outcome events (71.2%). Neither female sex (adjusted OR 1.09, 95% CI 0.69–1.72; p = 0.711) nor years of professional practice (adjusted OR 0.99, 95% CI 0.95–1.04; p = 0.779) was significantly associated with this outcome. Model discrimination was poor, with an AUC of 0.509 and Nagelkerke R² of 0.001, indicating that the included demographic and professional variables did not meaningfully explain variation in this perception.

Among dietitians, the model for perceived reduction of depressive symptoms included 282 observations and 186 outcome events (66.0%). Female sex showed a positive but non-significant association with this perception (adjusted OR 4.69, 95% CI 0.88–24.85; p = 0.069). Longer years of professional practice were associated with higher odds of perceiving GLP-1 RAs as reducing depressive symptoms (adjusted OR 1.08 per one-year increase, 95% CI 1.02–1.14; p = 0.010). This finding should be interpreted with caution because the number of male dietitians in the sample was very small. The model had an AUC of 0.623 and Nagelkerke R² of 0.055.

Among pharmacists, the model for perceiving GLP-1 RAs as very effective for WR based on patients’ feedback included 133 observations and 85 outcome events (63.9%). Female sex was not significantly associated with this perception (adjusted OR 0.69, 95% CI 0.32–1.48; p = 0.338). Longer years of professional practice were associated with lower odds of rating GLP-1 RAs as very effective for WR based on patients’ feedback (adjusted OR 0.93 per one-year increase, 95% CI 0.89–0.98; p = 0.011). The model had an AUC of 0.629 and Nagelkerke R² of 0.074.

Across all models, the Hosmer–Lemeshow goodness-of-fit tests did not indicate poor calibration. In the final models, VIF and tolerance values did not exceed the predefined thresholds for problematic collinearity. In physician models, age and years of professional practice were both retained despite elevated but acceptable VIF values. In non-physician models, age was omitted from the final models because initial diagnostics indicated substantial collinearity with years of professional practice.

Age and years of professional practice were strongly correlated in all professional groups (**Table 9**). Pearson correlation coefficients were r = 0.936 among physicians, r = 0.891 among nurses, r = 0.839 among physiotherapists, r = 0.991 among dietitians, and r = 0.958 among pharmacists; all p < 0.001. In the overall sample, the correlation between age and years of professional practice was r = 0.898; p < 0.001.

**Table 9:** Correlation and collinearity diagnostics for age and years of professional practice.

Professional group	N	Pearson r: age vs years of practice	p-value	Initial VIF for age	Initial VIF for years of practice	Initial tolerance for age	Initial tolerance for years of practice	Final modelling approach
Physicians	164	0.936	<0.001	8.21	8.20	0.122	0.122	Age and years retained
Nurses	160	0.891	<0.001	11.62	11.71	0.086	0.085	Age omitted; years retained
Physiotherapists	361	0.839	<0.001	17.59	17.56	0.057	0.057	Age omitted; years retained
Dietitians	282	0.991	<0.001	53.05	53.08	0.019	0.019	Age omitted; years retained
Pharmacists	133	0.958	<0.001	12.08	12.08	0.083	0.083	Age omitted; years retained
Overall sample	1,100	0.898	<0.001	—	—	—	—	Group-specific modelling applied

Pearson correlation coefficients were calculated separately for each professional group. Initial VIF and tolerance values refer to models in which both age and years of professional practice were considered. A VIF ≥10 or tolerance ≤0.10 was treated as indicating problematic collinearity. In physician models, both age and years of practice were retained because VIF values remained below the decision threshold of 10 applied during final model development, although elevated collinearity was acknowledged. In non-physician models, age was omitted from the final models and years of professional practice was retained because of its greater professional and clinical interpretability. VIF, variance inflation factor.

Collinearity diagnostics confirmed this relationship. In physician models, VIF for age and years of professional practice were elevated but remained below the decision threshold of 10 applied during final model development, with VIF values of 8.21 and 8.20, respectively, and tolerance values of 0.122 for both variables. Therefore, both variables were retained in the physician models.

In the initial non-physician models including both age and years of professional practice, problematic collinearity was observed. Among nurses, VIF values were 11.62 for age and 11.71 for years of practice, with tolerance values of 0.086 and 0.085, respectively. Among physiotherapists, VIF values were 17.59 and 17.56, with tolerance values of 0.057 for both variables. Among dietitians, VIF values were 53.05 and 53.08, with tolerance values of 0.019 for both variables. Among pharmacists, VIF values were 12.08 for both age and years of practice, with tolerance values of 0.083. Therefore, age was omitted from the final non-physician models, while years of professional practice was retained as the more professionally interpretable indicator of cumulative exposure.

Sex-related associations were examined as exploratory secondary findings. Unadjusted sex-stratified numbers and percentages are presented in **Table 10**, and full adjusted models are presented in **Table 1**.

**Table 10:** Unadjusted sex-stratified numbers and adjusted sex effects.

Professional group	Outcome	Female respondents with outcome, n/N (%)	Male respondents with outcome, n/N (%)	Adjusted sex effect from final model
Physicians	Very high effectiveness for weight loss	39/85 (45.9%)	47/79 (59.5%)	aOR 0.50, 95% CI 0.25-0.99; p = 0.046
Physicians	Perceived reduction of stress	57/85 (67.1%)	56/79 (70.9%)	aOR 0.83, 95% CI 0.42-1.64; p = 0.597
Physicians	Perceived reduction of anxiety	63/85 (74.1%)	50/79 (63.3%)	aOR 1.64, 95% CI 0.81-3.31; p = 0.171
Physicians	Perceived reduction of depressive symptoms	59/85 (69.4%)	58/79 (73.4%)	aOR 0.80, 95% CI 0.39-1.61; p = 0.526
Nurses	Perceived reduction of stress	50/134 (37.3%)	15/26 (57.7%)	aOR 0.54, 95% CI 0.22-1.30; p = 0.170
Physiotherapists	Perceived reduction of stress	144/200 (72.0%)	113/161 (70.2%)	aOR 1.09, 95% CI 0.69-1.72; p = 0.711
Dietitians	Perceived reduction of depressive symptoms	184/275 (66.9%)	2/7 (28.6%)	aOR 4.69, 95% CI 0.88-24.85; p = 0.069
Pharmacists	Very high effectiveness for weight loss based on patients’ feedback	49/79 (62.0%)	36/54 (66.7%)	aOR 0.69, 95% CI 0.32-1.48; p = 0.338

Sex-related associations were treated as exploratory secondary findings. Unadjusted numbers and percentages are presented by sex, together with the adjusted sex effect from the corresponding final multivariable model.

aOR, adjusted odds ratio; CI, confidence interval. Female sex was compared with male sex as the reference category. Adjusted estimates come from the final multivariable logistic regression models reported in **Table 1**. Sex-related analyses were exploratory; p-values should be interpreted cautiously in view of multiple testing and sex imbalance in selected professional groups.

Among physicians, very high perceived effectiveness of GLP-1 RAs for WR was reported by 39/85 female physicians, 45.9%, and 47/79 male physicians, 59.5%. In the adjusted model, female sex was associated with lower odds of reporting very high effectiveness compared with male sex, adjusted OR 0.50, 95% CI 0.25–0.99; p = 0.046. However, this association should be interpreted cautiously because it was exploratory and would not remain statistically significant after conservative correction for multiple testing.

For the remaining physician outcomes, no statistically significant adjusted sex-related associations were observed. Perceived stress reduction was reported by 57/85 female physicians, 67.1%, and 56/79 male physicians, 70.9%; adjusted OR 0.83, 95% CI 0.42–1.64; p = 0.597. Perceived anxiety reduction was reported by 63/85 female physicians, 74.1%, and 50/79 male physicians, 63.3%; adjusted OR 1.64, 95% CI 0.81–3.31; p = 0.171. Perceived reduction of depressive symptoms was reported by 59/85 female physicians, 69.4%, and 58/79 male physicians, 73.4%; adjusted OR 0.80, 95% CI 0.39–1.61; p = 0.526.

Among nurses, perceived stress reduction was reported by 50/134 female nurses, 37.3%, and 15/26 male nurses, 57.7%. Female sex was not significantly associated with this outcome in the adjusted model, adjusted OR 0.54, 95% CI 0.22–1.30; p = 0.170.

Among physiotherapists, perceived stress reduction was reported by 144/200 female physiotherapists, 72.0%, and 113/161 male physiotherapists, 70.2%. In the adjusted model, sex was not associated with perceived stress reduction, adjusted OR 1.09, 95% CI 0.69–1.72; p = 0.711. Therefore, no sex-related difference was identified among physiotherapists in the final model.

Among dietitians, perceived reduction of depressive symptoms was reported by 184/275 female dietitians, 66.9%, and 2/7 male dietitians, 28.6%. Female sex showed a positive but non-significant association in the adjusted model, adjusted OR 4.69, 95% CI 0.88–24.85; p = 0.069. This estimate should be interpreted with particular caution because the number of male dietitians was very small.

Among pharmacists, very high perceived effectiveness of GLP-1 RAs for WR based on patients’ feedback was reported by 49/79 female pharmacists, 62.0%, and 36/54 male pharmacists, 66.7%. Female sex was not significantly associated with this outcome in the adjusted model, adjusted OR 0.69, 95% CI 0.32–1.48; p = 0.338.

Overall, eight sex-related adjusted tests were performed across the final models. Given the exploratory nature of these analyses and the number of statistical tests performed, sex-related results were not interpreted as confirmatory findings. Under a conservative multiplicity perspective, no sex-related association remained robust.

## Discussion

### Principal findings and interpretation

The principal finding of this survey is that Polish HPs who participated in the study generally perceived GLP-1 RAs as effective and safe agents for cardiometabolic-risk management, while the strength and profile of these perceptions differed across professional groups. Physiotherapists and dietitians most frequently rated GLP-1 RAs as very effective for WR, whereas nurses more often selected uncertain or more cautious responses in several domains. Physicians more frequently reported perceived effects on HbA1c and lipid parameters, which may reflect their closer involvement in prescribing decisions and laboratory monitoring. Importantly, these results represent self-reported professional perceptions and should not be interpreted as objective knowledge scores or observed clinical outcomes in patients.

### Comparison with previous perception-based and implementation studies

The present findings are broadly consistent with earlier survey-based studies showing that GLP-1 RA implementation is influenced not only by clinical evidence, but also by professional role, familiarity with the drug class, prescribing experience, perceived safety, cost, access, and confidence in patient counselling. In a UK physician survey, Matza et al. reported positive perceptions of GLP-1 RAs, but also identified concerns related to injections, GI-AEs, and treatment burden (24). Similarly, surveys of CV and metabolic HPs have shown that lack of familiarity, limited prescribing confidence, resource constraints, prior authorization requirements, cost, and uncertainty about responsibility for initiation may restrict implementation of GLP-1 RAs and other glucose-lowering therapies with cardiometabolic benefit (25–30).

Compared with many previous studies, which focused primarily on physicians, prescribers, or selected specialties, our study included a broader multidisciplinary sample comprising physicians, pharmacists, nurses, physiotherapists, and dietitians. This may be particularly relevant for obesity and cardiometabolic-risk care, where patient education, treatment expectations, AE counselling, lifestyle support, and long-term adherence often depend on several professional groups rather than prescribers alone. However, because the sample was based on voluntary online participation, differences between professional groups should be interpreted cautiously and may also reflect recruitment channels, age and sex distribution, clinical setting, and prior exposure to GLP-1 RA-treated patients.

### Professional-group differences in perceptions

The particularly high recognition of WR efficacy among physiotherapists and dietitians may reflect their professional focus on lifestyle modification, functional improvement, and long-term patient support; however, the survey did not directly assess the reasons underlying participants’ responses. Therefore, this interpretation should be regarded as a plausible hypothesis rather than a causal explanation. Evidence from clinical studies indicates that sustained pharmacological WR can be associated with improvements in physical function, mobility, and quality of life (QoL), which may be especially relevant to professionals involved in lifestyle, rehabilitation, and nutritional care (31).

In contrast, physicians’ stronger focus on HbA1c and lipid-related effects may reflect clinical monitoring priorities and direct responsibility for pharmacological treatment decisions. This observation is consistent with prior research showing that prescribers and clinicians responsible for metabolic monitoring tend to prioritize laboratory endpoints, whereas other professional groups focus more on functional, behavioural, and patient-reported outcomes (25–30, 32, 33). The more cautious responses among nurses could indicate differences in clinical exposure, training opportunities, or the type of patient interactions encountered in daily practice, although these explanations were not directly tested in the present survey.

### Perceived psychological and behavioural effects

Perceptions concerning stress, anxiety, depressive symptoms, smoking, and alcohol consumption require especially careful interpretation. The questionnaire assessed whether HPs perceived such effects in relation to GLP-1 RA therapy; it did not measure psychiatric symptoms, alcohol use, or smoking behaviour in patients. Therefore, these findings should be understood as professional beliefs or observations from clinical practice rather than evidence that GLP-1 RAs improve mental health or reduce substance use. The relatively high endorsement of psychological and behavioural effects may have been influenced by emerging scientific reports and public discussion regarding reward pathways, appetite regulation, and patient-reported experiences during GLP-1 RA treatment (34–37). At the same time, the substantial proportion of uncertain responses in some groups suggests that many respondents recognised the limited and still evolving nature of this evidence.

### Safety, access, and educational implications

The generally favourable safety perceptions observed in this survey are consistent with the established tolerability profile of GLP-1 RAs, in which GI symptoms are the most commonly recognised AEs (38–41). However, the present study did not assess actual adverse-event rates, clinical monitoring, or treatment discontinuation. Support for reimbursement was high across professional groups, which could indicate that respondents perceived access and affordability as important issues in clinical practice. This interpretation is consistent with previous implementation studies identifying cost, insurance or reimbursement restrictions, administrative burden, and limited prescriber confidence as barriers to broader use of GLP-1 RAs and related cardiometabolic therapies (26, 27, 30).

Overall, the findings suggest that interdisciplinary education may be useful, particularly if it is tailored to professional roles. For prescribers, education may focus on indications, contraindications, CV and metabolic evidence, and monitoring. For nurses, pharmacists, dietitians, and physiotherapists, training may emphasize realistic counselling, AE recognition, lifestyle support, adherence, and referral pathways. Because this survey assessed perceptions rather than educational outcomes, these implications should be viewed as directions for future implementation work rather than conclusions about proven educational deficits.

Sex-related estimates were treated as exploratory. Although sex was included as an adjustment covariate, the study was not designed or powered to test sex differences in perceptions of GLP-1 RAs. Moreover, the distribution of sex was markedly imbalanced in some professional groups, particularly among dietitians and nurses, which limits the precision and interpretability of sex-related estimates. Several p-values were close to 0.05 and no sex-related association remained robust under a conservative multiplicity perspective. Therefore, these findings should not be interpreted as confirmatory evidence of sex-based differences, but rather as descriptive and hypothesis-generating observations.

### Current cardiovascular evidence and clinical heterogeneity

The participating HPs’ generally favourable assessment of cardiometabolic value is broadly aligned with contemporary cardiovascular evidence, but GLP-1 RA benefit should not be interpreted as identical across agents, outcomes, or patient phenotypes. A 2025 systematic review and meta-analysis identified baseline age and body mass index as statistically significant modifiers of cardiovascular benefit, suggesting that the relative reduction in major adverse cardiovascular events may vary according to clinical profile (9). A subsequent synthesis of long-term cardiovascular outcome trials in high-risk populations reported reductions in major adverse cardiovascular events, cardiovascular and all-cause mortality, non-fatal myocardial infarction, non-fatal stroke, and hospitalization for heart failure, without a material increase in severe hypoglycaemia or acute pancreatitis; gastrointestinal adverse effects remained more frequent (22). Accordingly, professional education should move beyond a simplified class-benefit message and integrate baseline cardiovascular risk, comorbidities, treatment goals, tolerability, and expected persistence into shared decision-making.

### Real-world effectiveness, persistence, mortality, and implementation gaps

Randomized trials establish efficacy under controlled conditions, whereas real-world studies clarify whether benefits are retained during routine initiation, continuation, interruption, or discontinuation. In patients with T2DM at increased cardiovascular risk, sustained GLP-1 RA use was associated with lower risks of three-point major adverse cardiovascular events, cardiovascular and all-cause mortality, heart failure, and unstable angina compared with sustained DPP-4 inhibitor use; no significant differences were reported for myocardial infarction or stroke (10). In the CARDIAB cohort, use of either SGLT2 inhibitors or GLP-1 RAs was associated with lower observed mortality among patients with diabetes and cardiovascular disease, although the observational design does not establish causality; uptake remained low, particularly among women, older patients, individuals with non-coronary atherosclerotic disease, and those not followed in specialist clinics (11). The distinction between efficacy and effectiveness is especially relevant to this survey because population benefit depends on equitable access, appropriate initiation, adherence, persistence, dose escalation, adverse-effect management, and continuity of multidisciplinary care. Positive professional perceptions should therefore not be assumed to translate automatically into guideline-concordant prescribing or sustained exposure.

### GLP-1 RAs and SGLT2 inhibitors in heart failure, frailty, and cerebrovascular care

GLP-1 RAs and SGLT2 inhibitors should be viewed as complementary rather than universally interchangeable cardiometabolic therapies. In patients with diabetes and established cardiovascular disease, observational data associate both classes with lower mortality, but their principal clinical strengths differ (11). SGLT2 inhibitors have the most established role in reducing heart-failure hospitalization and slowing chronic kidney disease progression, whereas GLP-1 RAs have particularly consistent effects on weight and atherosclerotic cardiovascular outcomes. In diabetic HFpEF, SGLT2 inhibitors remain foundational outcome-modifying therapy, while GLP-1-based agents may add value in obesity-associated disease through weight reduction, improved metabolic control, and potential effects on vascular and myocardial biology (18). Multiomics analyses implicate inflammatory signalling, oxidative stress, endothelial dysfunction, lipid metabolism, mitochondrial pathways, and cardiac remodelling, but these mechanistic findings require confirmation in phenotype-specific prospective studies (19). For frail older adults, neither class can be labelled universally superior; treatment selection should account for renal function, volume status, nutritional status, polypharmacy, gastrointestinal and genitourinary adverse effects, and the risk of worsening sarcopenia (20). Recent utilization data among patients with ischaemic stroke also show increasing but uneven use of both classes, identifying a secondary-prevention implementation gap (21). Thus, the broadly positive perceptions recorded in this survey should be interpreted within a comorbidity-driven treatment-selection framework rather than as support for uniform preference for one drug class.

### Oral GLP-1 therapy and orforglipron

The transition from injectable peptides to oral formulations may change access, treatment acceptability, and professional perceptions. Oral GLP-1 therapy can reduce injection-related barriers and may broaden uptake in obesity and T2DM, although adherence will still depend on tolerability, affordability, dosing instructions, and continuity of care (12). Orforglipron is a once-daily, non-peptide small-molecule GLP-1 receptor agonist that can be taken without the fasting and water restrictions required for oral semaglutide. Phase 2 and phase 3 studies have demonstrated clinically meaningful reductions in body weight and HbA1c, while analyses in participants with T2DM or obesity without diabetes have reported improvements in systolic blood pressure, lipid measures, and other cardiovascular risk biomarkers (13–15). A recent systematic review and meta-analysis similarly found favourable cardiometabolic efficacy with a safety profile dominated by gastrointestinal adverse effects, particularly during dose escalation (16). These findings support the promise of orforglipron as an oral option; however, biomarker improvement and weight loss cannot be assumed to reduce cardiovascular events. Dedicated cardiovascular and renal outcome trials, longer follow-up, comparative-effectiveness research, and post-marketing surveillance remain necessary before newer oral small molecules can be considered equivalent to agents with completed outcome trials.

### Evidence beyond T2DM and emerging safety signals

The widening therapeutic landscape also requires careful boundary setting. In T1DM, adjunctive GLP-1 agonist therapy has been associated with modest reductions in HbA1c and total daily insulin dose, but the evidence is heterogeneous and does not support replacement of insulin (17). Any adjustment of insulin dose should therefore be individualized and undertaken under specialist supervision. Safety discussions should also extend beyond the gastrointestinal and injection-site effects most frequently recognised by respondents. Long-term trial synthesis supports an overall favourable cardiovascular safety profile in high-risk populations (22), yet uncommon, delayed, and population-specific events require continued pharmacovigilance. A recent systematic review found pulmonary adverse-event signals to be uncommon and heterogeneous, with uncertainty regarding causality; delayed gastric emptying may also be relevant to peri-procedural aspiration risk, although the available evidence remains limited (23). The high perceived safety recorded in this survey should therefore be interpreted as a favourable overall view, not as evidence that respondents recognise all uncommon risks, drug-specific warnings, or circumstances requiring treatment interruption.

### Implications for interpretation of the survey

Taken together, the current evidence strengthens the clinical rationale for the favourable perceptions observed in this study while demonstrating why perceptions alone cannot serve as a proxy for evidence-based competence. Clinical modifiers and long-term outcome data support phenotype-specific selection rather than uniform class-based prescribing (9, 22). Real-world evidence emphasizes persistence, equitable access, and comorbidity-driven complementary use of GLP-1 RAs and SGLT2 inhibitors (10, 11). Oral therapies may reduce injection-related barriers, but newer small molecules do not yet have completed cardiovascular outcome trials despite favourable effects on body weight, HbA1c, and cardiovascular risk biomarkers (12–16). Evidence relating to T1DM, HFpEF, multiomics, frailty, post-stroke prescribing, and pulmonary safety broadens the educational agenda but does not establish universal indications or a single preferred therapy (17–21, 23). Thus, the high perceived effectiveness, safety, and reimbursement support observed in this survey should be regarded as a favourable foundation for implementation, but not as evidence of sufficiently current and outcome-specific knowledge across all rapidly evolving areas. Future Polish studies should assess objective knowledge, guideline-concordant prescribing and referral, treatment persistence, affordability, reasons for discontinuation, and cardiovascular-risk-based treatment selection.

### Practical implications

From a practical perspective, the findings support structured, profession-specific education on incretin-based therapies. Prescribers require current knowledge of indications, contraindications, cardiovascular and renal outcome evidence, patient selection, combination strategies, titration, and long-term monitoring. Nurses and pharmacists can reinforce administration, persistence, adverse-effect management, and referral or escalation pathways, whereas dietitians and physiotherapists are central to preserving nutritional quality, lean mass, physical function, and realistic expectations during pharmacological weight reduction. Educational activities should also address management of gastrointestinal symptoms, hypoglycaemia risk when GLP-1 RAs are combined with insulin or sulfonylureas, weight regain after discontinuation, and the evidence gaps surrounding newer oral agents. Future implementation research should evaluate whether structured education improves objective knowledge, referral and prescribing behaviour, treatment persistence, and patient outcomes. Mechanistic and multiomics findings should be presented as research directions rather than established clinical indications.

### Limitations

Several limitations should be considered. First, the study used non-probability convenience sampling; therefore, the findings are not statistically representative of all healthcare professionals in Poland. Although the final sample exceeded the number required for a nominal margin of error of ±3 percentage points at the 95% confidence level, that calculation assumes simple random sampling and does not establish population representativeness. The eligibility criterion requiring familiarity with, or professional exposure to, incretin-based therapies may have selected respondents who were already more interested in, more familiar with, or more positively disposed towards GLP-1 RAs. Unequal representation of professional groups and differences in recruitment channels, age and sex structure, clinical setting, professional experience, and exposure to GLP-1 RA therapy may also have contributed to the observed between-group differences; these differences should not be attributed to professional role alone. No regional sampling frame, voivodeship-level quotas, or stratified recruitment strategy was used, and respondent-level regional data were unavailable for a complete assessment of geographical coverage.

Second, the study-specific questionnaire was reviewed internally for content relevance, clarity, comprehensibility, and face validity, but it was not a previously validated psychometric instrument. No formal pilot testing involving the target population, quantitative content-validity assessment, construct validation, exploratory factor analysis, or reliability analysis was performed. Because the questionnaire did not generate a total score or composite scale and the heterogeneous categorical items were analysed individually, the findings should be interpreted as self-reported perceptions and professional views rather than objectively measured knowledge or clinical competence.

Third, because the survey link was disseminated through multiple professional and institutional channels and no unique invitation or visitor log was available, the sampling denominator and the response, participation, and completion rates could not be calculated. The available export contained complete submitted questionnaires retained for eligibility and data-quality screening, but the numbers of unique visitors, questionnaire starts, abandoned questionnaires, and incomplete pre-submission records were unavailable. In addition, because direct identifiers, IP addresses, cookies, and login-based identifiers were not retained, duplicate detection relied on demographic, professional, and response-pattern similarity. This approach protected anonymity but could not completely exclude duplicate submissions.

An additional limitation is the exceptionally rapid evolution of the GLP-1 field. Several publications requested during editorial review were released after data collection, including reports on oral small-molecule agonists, long-term cardiovascular outcomes, pulmonary safety signals, and prescribing after ischaemic stroke (15, 16, 21–23). Respondents could not have been expected to incorporate evidence that was unavailable when the survey was completed. The present revision therefore uses these studies to contextualize the findings and define future educational needs, not to retrospectively judge participants against later evidence.

Fourth, psychological, smoking-related, and alcohol-related outcomes were not assessed using validated clinical instruments and should be interpreted as perceived effects rather than patient-level clinical outcomes. The regression models were exploratory; continuous predictors were modelled per one-year increase, no formal interaction analysis was performed, and influential observations were not assessed separately. Multiple comparisons further require cautious interpretation of associations close to p = 0.05. Finally, because the study was conducted in Poland, the findings may not be directly generalisable to healthcare systems with different reimbursement policies, access pathways, or professional roles.

## Conclusions

This cross-sectional online survey showed that participating HPs in Poland generally perceived GLP-1 RAs as effective and safe pharmacological options in cardiometabolic risk management. Respondents most consistently recognised their role in WR and selected metabolic outcomes, while perceptions of psychological and behavioural effects varied across professional groups. Support for reimbursement was also high among the surveyed participants.

However, the findings should be interpreted as self-reported perceptions of a non-probability sample of HPs with prior familiarity with, or professional exposure to, incretin-based therapies. The observed differences between professional groups should not be attributed to professional role alone, as they may also reflect recruitment channels, clinical setting, professional experience, demographic structure, and degree of exposure to GLP-1 RA therapy.

The rapidly evolving evidence base underscores the need for continuously updated, profession-specific education that distinguishes established cardiovascular outcome evidence from emerging mechanistic, oral-therapy, and safety data. Further studies using dedicated instruments and more representative sampling should evaluate objective knowledge, prescribing and referral behaviour, access barriers, treatment persistence, adverse-effect management, and the contribution of interdisciplinary care to guideline-concordant cardiometabolic treatment.

## Data Availability

The original contributions presented in the study are included in the article/**Supplementary Material**. Further inquiries can be directed to the corresponding author.
