# Supplementary material for: Polish healthcare professionals’ perceptions of GLP-1 receptor agonists in cardiometabolic risk reduction: a cross-sectional online survey in Poland
Source: Front Endocrinol (Lausanne). 2026 Aug 4;17:1906959. doi: 10.3389/fendo.2026.1906959 (PMC13481303; doi:10.3389/fendo.2026.1906959)
Supplement: Supplementary file 1 [file Table1.docx]

Supplementary Materials

*Polish Healthcare Professionals’ Perceptions of GLP-1 Receptor Agonists in Cardiometabolic Risk Reduction: A Cross-Sectional Online Survey in Poland*

# Supplementary Material 1. STROBE checklist for cross-sectional studies

| **Section** | **Item** | **STROBE reporting recommendation** | **Location and study-specific reporting** |
| --- | --- | --- | --- |
| **Title and abstract** | **1(a)** | Identify the study design using a standard term in the title or abstract. | Title; Abstract - Design. The study is described as a cross-sectional online survey conducted among healthcare professionals in Poland. |
| **Title and abstract** | **1(b)** | Provide an informative and balanced abstract summarising what was done and what was found. | Abstract - Background, Objectives, Design, Methods, Results and Conclusions. Numerical findings, adjusted odds ratios, 95% confidence intervals and p-values are included for selected principal results. |
| **Introduction** | **2** | Explain the scientific background and rationale for the study. | Introduction. The revised text summarises cardiometabolic relevance, previous surveys, implementation-related evidence and the multidisciplinary research gap. |
| **Introduction** | **3** | State the study objectives and any prespecified hypotheses. | End of Introduction. The objective is to assess healthcare professionals’ perceptions of GLP-1 receptor agonists and related incretin-based therapies in cardiometabolic risk management. No formal prespecified hypothesis is reported. |
| **Methods** | **4** | Present the main elements of the study design early in the Methods section. | Materials and methods - Study design and reporting. |
| **Methods** | **5** | Describe the setting, locations and relevant dates, including the recruitment and data-collection period. | Study design and reporting; Recruitment and geographical scope. The survey was available across Poland and was conducted from May to August 2025. |
| **Methods** | **6(a)** | Describe eligibility criteria and the sources and methods used to select participants. | Recruitment and sampling - Inclusion criteria, Exclusion criteria, and Recruitment and geographical scope. Non-probability convenience sampling was used through professional, institutional, academic and online professional channels. |
| **Methods** | **7** | Define outcomes, exposures, predictors, potential confounders and effect modifiers. | Questionnaire development, content and response options; Statistical analysis; Table 8. Outcomes are self-reported perceptions, not objectively measured clinical effects or knowledge scores. Predictor and reference-category coding is provided for multivariable models. |
| **Methods** | **8** | For each variable, report the data source and assessment method and explain comparability across groups. | Questionnaire development, content and response options; Questionnaire validation and psychometric characteristics; Supplementary Material 4. A study-specific questionnaire was used. Items were reviewed by the multidisciplinary research team for content relevance, clarity and face validity. The questionnaire did not generate a total or domain score, and no full psychometric construct validation or internal-consistency analysis was performed. |
| **Methods** | **9** | Describe measures taken to address potential sources of bias. | Recruitment and geographical scope; Duplicate and data-quality control; Discussion - Limitations. The manuscript addresses convenience sampling, self-selection, eligibility based on prior familiarity or exposure, unequal professional-group representation, self-reporting, unavailable response metrics, residual duplicate risk and limited generalisability. |
| **Methods** | **10** | Explain how the study size was determined. | Methods - Sample-size rationale. No formal a priori sample-size calculation was performed. Recruitment was conducted during the predefined survey period. A retrospective nominal-precision calculation showed that approximately 1,068 respondents were required to estimate a proportion with a maximum margin of error of ±3 percentage points at the 95% confidence level, assuming p = 0.50 and simple random sampling. The final analytical sample included 1,100 respondents. This calculation does not establish population representativeness because non-probability convenience sampling was used. |
| **Methods** | **11** | Explain how quantitative variables were handled in analysis, including any categorisation. | Missing data and statistical handling; Normality and collinearity diagnostics; Statistical analysis; Tables 1, 8 and 9. Age and years of practice were analysed as continuous variables and entered into regression models per one-year increase. Categorical outcomes were analysed using their original categories or binary coding shown in Table 8. |
| **Methods** | **12(a)** | Describe all statistical methods, including methods used to control for confounding. | Statistical analysis. Categorical comparisons used chi-square or Fisher’s exact tests, with Cramer’s V where appropriate. Multivariable logistic regression reported adjusted odds ratios, 95% confidence intervals and p-values. Model fit and collinearity diagnostics were reported. |
| **Methods** | **12(b)** | Describe methods used to examine subgroups and interactions. | Statistical analysis; Tables 8 and 9; Table 10. Analyses were stratified by professional group where specified. Sex-related analyses were treated as secondary and exploratory. No formal interaction analysis was reported. |
| **Methods** | **12(c)** | Explain how missing data were handled. | Missing data and statistical handling. The final analytical export contained 1,100 complete and eligible submitted questionnaires. Analyses used valid observations for each variable, and multivariable models used complete-case analysis for the variables included in each model. Valid n is reported where applicable. |
| **Methods** | **12(d)** | If applicable, explain analytical methods accounting for the sampling strategy. | Recruitment and geographical scope; Statistical analysis; Limitations. The study used non-probability convenience sampling. No sampling weights, post-stratification or design-based corrections were applied. |
| **Methods** | **12(e)** | Describe any sensitivity analyses. | No formal sensitivity analysis was reported. Collinearity was assessed during model development, and age was omitted from final non-physician models when collinearity with years of practice was substantial. |
| **Results** | **13(a)** | Report participant numbers at each stage, including those assessed, eligible, included and analysed. | Results - Characteristics of the study group; Supplementary Material 3. The final analytical sample comprised 1,100 respondents: physicians n = 164, pharmacists n = 133, nurses n = 160, physiotherapists n = 361 and dietitians n = 282. |
| **Results** | **13(b)** | Give reasons for non-participation or exclusion at each stage. | Supplementary Material 3. The exact numbers of people reached, survey starts, incomplete pre-submission records and exclusions before the final export were not retained and cannot be reconstructed. Eligibility, completeness, plausibility, internal consistency and duplicate-like patterns were assessed before final analysis. |
| **Results** | **13(c)** | Consider presenting participant flow in a diagram or structured summary. | Supplementary Material 3 - Participant flow and data-quality screening summary. |
| **Results** | **14(a)** | Report participant characteristics and relevant exposures or potential confounders. | Table 1. Professional group, sex, age and years of professional practice are reported. |
| **Results** | **14(b)** | Report the number of participants with missing data for variables of interest. | Tables and model summaries report valid analytical n where applicable. The final export contained complete submitted questionnaires; complete-case n and event counts are reported for each multivariable model in Table 8. |
| **Results** | **15** | Report outcome-event numbers or summary measures. | Tables 2-7 report frequencies and percentages for perception-based outcomes. Table 8 reports analytical sample size and number and percentage of outcome events for each logistic regression model. |
| **Results** | **16(a)** | Report unadjusted and, where applicable, adjusted estimates with measures of precision; identify adjustment variables. | Tables 2-9 and Table 10. Between-group comparisons, effect sizes, adjusted odds ratios, 95% confidence intervals, exact p-values, variables entered and model-fit statistics are reported. |
| **Results** | **16(b)** | Report category boundaries when continuous variables are categorised. | Table 8 provides complete outcome and reference-category coding. Continuous age and years of practice were modelled per one-year increase rather than categorised. |
| **Results** | **16(c)** | If relevant, translate relative estimates into absolute risk over a meaningful period. | Not applicable. The study was cross-sectional and assessed perceptions rather than prospective clinical risk. |
| **Results** | **17** | Report other analyses, such as subgroup, interaction or sensitivity analyses. | Profession-stratified analyses, exploratory sex-related analyses and collinearity diagnostics are reported in Tables 8-9 and Table 10. No formal interaction or sensitivity analyses were reported. |
| **Discussion** | **18** | Summarise the main results in relation to the study objectives. | Discussion - opening and key-results paragraphs. |
| **Discussion** | **19** | Discuss limitations, including potential bias and imprecision and their likely effect on interpretation. | Discussion - Limitations. The section addresses convenience sampling, self-selection, prior familiarity/exposure, unequal group composition, unavailable denominator and response metrics, non-validated perception measures, residual duplicate risk, multiple testing and limited external validity. |
| **Discussion** | **20** | Provide a cautious overall interpretation considering objectives, limitations, multiplicity and other evidence. | Discussion and Conclusions. Findings are explicitly interpreted as self-reported perceptions and not as causal or objectively measured clinical effects. Exploratory and multiple-comparison findings are interpreted cautiously. |
| **Discussion** | **21** | Discuss generalisability or external validity. | Discussion - Limitations and Conclusions. Findings apply to participating healthcare professionals in Poland and are not presented as representative estimates for the entire Polish healthcare workforce or other healthcare systems. |
| **Other information** | **22** | State the source of funding and the role of funders. | Funding statement. No external funding was reported. |

*Reference: von Elm E, Altman DG, Egger M, et al. The Strengthening the Reporting of Observational Studies in Epidemiology (STROBE) Statement: guidelines for reporting observational studies.*

# Supplementary Material 2. CHERRIES reporting addendum for the online survey

| **CHERRIES category** | **Checklist item** | **What should be reported** | **Study-specific reporting** |
| --- | --- | --- | --- |
| **Design** | **Survey design** | Describe the target population, sampling frame and whether the sample was a convenience sample. | Anonymous cross-sectional online survey of licensed healthcare professionals in Poland. Non-probability convenience sampling was used. The final sample is not considered geographically or workforce representative. |
| **Ethics and consent** | **Ethics approval** | State whether ethics approval was obtained. | Approved by the Bioethics Committee of Poznan University of Medical Sciences, Poland (approval no. KB-301/25; 14 May 2025). The study was conducted in accordance with the Declaration of Helsinki. |
| **Ethics and consent** | **Informed consent** | Describe the information provided and the consent mechanism. | Before entering the questionnaire, potential participants received information on the study purpose, voluntary participation, anonymity, eligibility and the scope of the survey. Submission of the completed questionnaire was treated as informed consent. |
| **Ethics and consent** | **Data protection** | Describe safeguards for personal information and unauthorised access. | No names, telephone numbers, e-mail addresses, workplace identifiers, patient-level data or other direct personal identifiers were included in the analytical dataset. IP addresses, cookies and login-based identifiers were not used as mandatory respondent identifiers in the analytical dataset. |
| **Development and pre-testing** | **Development and testing** | Explain how the survey was developed and whether usability and technical functionality were tested before fielding. | The study-specific questionnaire was developed from current clinical and pharmacological knowledge, the study objectives, and the research team’s expertise. The draft was reviewed internally by a multidisciplinary team for content relevance, clarity, comprehensibility, and face validity, and individual items were revised accordingly. No quantitative content-validity index was calculated. The questionnaire was not an objective knowledge test and did not generate a total score or composite scale. No formal pilot testing involving the target population, construct validation, exploratory factor analysis, or Cronbach’s alpha analysis was performed. Formal usability or technical pre-testing was not documented in the available study records. |
| **Recruitment** | **Open versus closed survey** | State whether the survey was open to all visitors or restricted to a known sample. | The survey used an open web link disseminated through professional channels. Access to the link was technically open, but eligibility was restricted to licensed healthcare professionals who met the predefined inclusion criteria. |
| **Recruitment** | **Contact mode** | State whether the initial contact was made online or offline. | Potential participants were contacted electronically through professional, institutional, university, clinical, academic and professional online communication channels. |
| **Recruitment** | **Advertising the survey** | Describe how and where the survey was announced and provide sufficient context to understand self-selection. | The link was distributed through healthcare professional networks, institutional and university channels, clinical and academic contacts, and professional online groups in Poland. A central invitation log and profession-specific invitation denominators were not available. Recruitment wording and a complete list of dissemination channels were not retained in a form permitting full reconstruction. |
| **Survey administration** | **Web/e-mail mode** | Describe the type of electronic survey and how responses were captured. | The questionnaire was self-administered online through a web link, and submitted responses were exported electronically for analysis. The platform name was not documented in the available study records. |
| **Survey administration** | **Recruitment context** | Describe the website, mailing list or other setting in which the survey was presented and how this context may have influenced selection. | The survey was accessed through a direct web link distributed within professional and institutional networks rather than through one public general-interest website. This recruitment context may have preferentially reached professionals with greater interest in obesity, diabetes, cardiometabolic prevention or incretin-based therapies. |
| **Survey administration** | **Mandatory or voluntary participation** | State whether completion was mandatory or voluntary. | Participation was entirely voluntary. Eligible recipients could choose whether to open and submit the questionnaire. |
| **Survey administration** | **Incentives** | Report any monetary, material or non-monetary incentives. | No financial, material or other incentives were offered. |
| **Survey administration** | **Time and date** | State the data-collection timeframe. | Data were collected from May to August 2025. Recruitment ended after the predefined survey period; no formal statistical stopping rule was used. |
| **Survey administration** | **Randomisation of items or questionnaires** | State whether item order or questionnaire versions were randomised. | Randomisation of items or questionnaire versions was not documented in the available study records. |
| **Survey administration** | **Adaptive questioning** | Describe branching or conditional display of items. | The questionnaire contained profession-related content, but the electronic branching or skip-logic settings were not documented in the available study records. No unsupported claim about adaptive questioning is made. |
| **Survey administration** | **Number of items** | Report the number of questionnaire items shown to respondents. | The exact item count will be reported with the complete questionnaire in Supplementary Material 4. An unsupported item count is not reported in the current addendum. |
| **Survey administration** | **Number of screens/pages** | Report how many electronic pages or screens were used. | The number of electronic screens/pages was not retained in the available study records and cannot be reported reliably. |
| **Survey administration** | **Completeness check** | State whether mandatory-item or consistency checks were applied before submission. | The final analytical export contained complete submitted questionnaires. The platform-level configuration of mandatory fields and real-time completeness or consistency checks was not documented. Post-export screening assessed eligibility, completeness, plausibility, internal consistency and duplicate-like patterns. |
| **Survey administration** | **Review step** | State whether respondents could review and change answers before final submission. | The availability of a Back button or final review screen was not documented in the available platform records. |
| **Response metrics** | **Unique site visitor** | Explain how unique visitors were defined if response metrics are reported. | Unique visitors were not recorded. Direct technical identifiers were not retained in the analytical dataset. |
| **Response metrics** | **View rate** | Report unique survey visitors divided by unique site visitors, if calculable. | Not calculable. Landing-page views and unique site visitors were not recorded. |
| **Response metrics** | **Participation rate** | Report the number agreeing to participate divided by unique first-page visitors, if calculable. | Not calculable. Unique first-page visitor and questionnaire-start counts were not available. |
| **Response metrics** | **Completion rate** | Report the number finishing the survey divided by those who agreed or started, if calculable. | Not calculable. The number of starts or consent-page completions was not available. The final analytical export contained 1,100 complete and eligible submitted questionnaires. |
| **Preventing multiple entries** | **Cookies** | State whether cookies were used to identify or prevent repeated submissions. | Cookies were not used as mandatory respondent identifiers in the analytical dataset. The exact platform-level cookie configuration was not documented. |
| **Preventing multiple entries** | **IP check** | State whether IP addresses were used to identify repeated submissions. | IP addresses were not retained or used as mandatory respondent identifiers in the analytical dataset in order to protect anonymity. |
| **Preventing multiple entries** | **Log-file analysis** | Describe any other log-file methods used to identify repeated submissions. | No log-file-based duplicate-identification procedure was documented. Duplicate-like records were assessed after export using combinations of demographic/professional characteristics and identical or near-identical response patterns. |
| **Preventing multiple entries** | **Registration/login** | For closed surveys, describe registration or login controls. | Not applicable. The survey was not password-protected and did not require registration or login. |
| **Analysis** | **Handling incomplete questionnaires** | State whether only complete questionnaires were analysed and how early terminations were handled. | Only complete submitted questionnaires retained in the final analytical export were analysed. Incomplete or abandoned pre-submission records were not available in the export; their exact number cannot be reconstructed. |
| **Analysis** | **Atypical completion time** | Describe any exclusion rule based on unusually short or long completion time. | No exclusion criterion based on completion time was reported, and completion-time data were not available for retrospective assessment. |
| **Analysis** | **Statistical correction for non-representativeness** | Describe weighting, propensity-score adjustment or other corrections for a non-representative sample. | No weighting, propensity-score adjustment or post-stratification was applied. Results are interpreted as perceptions of participating healthcare professionals rather than population-representative estimates. |

*Reference: Eysenbach G. Improving the Quality of Web Surveys: The Checklist for Reporting Results of Internet E-Surveys (CHERRIES). J Med Internet Res. 2004;6(3):e34.*

# Supplementary Material 3. Participant flow and data-quality screening summary

| **Flow stage** | **Number/status** | **Transparent reporting statement** |
| --- | --- | --- |
| **Potential healthcare professionals reached or invited** | **Not determinable** | The open survey link was distributed through multiple professional, institutional, university, clinical, academic and online professional channels. No central invitation denominator was maintained. |
| **Survey-link or landing-page views** | **Not recorded** | Unique visitor and landing-page analytics were not available; the view rate cannot be calculated. |
| **Questionnaire starts or consent-page completions** | **Not recorded** | The survey platform/export did not preserve a reliable count of questionnaire starts or consent-page completions; participation and completion rates cannot be calculated. |
| **Incomplete or abandoned pre-submission questionnaires** | **Exact number unavailable** | Incomplete or abandoned records were not retained in the final analytical export. Their number cannot be retrospectively reconstructed and is not assumed to be zero. |
| **Submitted records available in the final analytical export** | **1,100 complete and eligible questionnaires** | The final analytical export comprised complete submitted questionnaires that met the eligibility and data-quality criteria. |
| **Records excluded as ineligible before the final analytical dataset** | **Exact pre-export number unavailable** | Eligibility was assessed before final analysis. The available records do not permit reconstruction of a separate numerical count of pre-export ineligible submissions. |
| **Records excluded for implausible, internally inconsistent or duplicate-like patterns** | **Exact pre-export number unavailable; none retained in the final analytical dataset** | Potentially problematic records were flagged using predefined logical and plausibility rules and reviewed manually. The available export does not permit reliable reconstruction of category-specific exclusion counts. |
| **Final analytical sample** | **1,100** | All records retained for analysis were complete, eligible and met the stated data-quality criteria. |
| **Physicians** | **n = 164** | Final professional-group distribution. |
| **Pharmacists** | **n = 133** | Final professional-group distribution. |
| **Nurses** | **n = 160** | Final professional-group distribution. |
| **Physiotherapists** | **n = 361** | Final professional-group distribution. |
| **Dietitians** | **n = 282** | Final professional-group distribution. |

## Data-quality screening criteria and decision rules

| **Screening component** | **Operational definition and procedure** |
| --- | --- |
| **Eligibility** | **Eligible professions were physicians, pharmacists, nurses, physiotherapists and dietitians. Participants were required to be in active clinical or professional practice, involved in care related to type 2 diabetes, obesity or cardiovascular disease, and familiar with or professionally exposed to incretin-based therapies.** |
| **Completeness** | **The final analytical dataset included complete submitted questionnaires. Incomplete or abandoned pre-submission records were not available in the final export.** |
| **Implausible age** | **Age below 22 years or above 80 years was considered implausible for the target population and was flagged for manual review.** |
| **Implausible professional experience** | **Years of professional practice below 0 or above 60 years were considered implausible. Years of practice exceeding age minus 18 years were flagged as logically implausible and manually reviewed.** |
| **Internally inconsistent response pattern** | **Examples included an ineligible professional category combined with completion of profession-specific sections; reporting no active professional practice despite other eligibility responses; mutually incompatible professional role and work-setting responses; years of practice incompatible with age; contradictory answers to related clinical-exposure items; or non-differentiated responding that conflicted with filter or profession-specific questions.** |
| **Suspected duplicate record** | **A record was considered duplicate-like only when identical or near-identical combinations of professional group, sex, age, years of practice and specialist work setting occurred together with identical or near-identical responses across the main questionnaire items. Demographic similarity alone was not sufficient.** |
| **Screening procedure** | **Screening was conducted in two stages: automated or rule-based flagging of potentially problematic records, followed by manual review before any exclusion decision.** |
| **Decision rule** | **A record was excluded only when the available information provided clear evidence of ineligibility, implausibility, internal inconsistency or suspected duplication. No record was excluded solely because of one unusual but plausible answer.** |
| **Ambiguous cases** | **Ambiguous cases were resolved conservatively. If a record could not be confidently classified as invalid, it was retained.** |
| **Anonymity and duplicate control** | **Direct personal identifiers were not collected. IP addresses, cookies and login-based identifiers were not used as mandatory respondent identifiers in the analytical dataset. Consequently, duplicate submissions could not be ruled out completely; response-pattern and plausibility screening was used to reduce this risk.** |
| **Recruitment stopping rule** | **No formal statistical stopping rule was prespecified. Recruitment was conducted during the predefined period from May to August 2025 and ended when that period closed.** |

**Supplementary Material 4 – Complete study questionnaire**.

# Clinical and Social Aspects of the Use of the Latest Pharmacotherapy for Obesity and/or Type 2 Diabetes

## Information for Participants

Dear Sir/Madam,

This questionnaire has been developed for use in the doctoral dissertation entitled:
"Clinical and Social Aspects of the Use of the Latest Pharmacotherapy for Obesity and/or Type 2 Diabetes."

The aim of this study is to evaluate the practical application of the latest incretin hormone analogues in preventing selected cardiovascular risk factors, including obesity, type 2 diabetes mellitus, depressive disorders, alcohol dependence, and nicotine dependence.

If you are a physician currently practicing in Poland, you are invited to participate in this study.

Completion of the questionnaire will take approximately 35–40 minutes. Participation is voluntary and anonymous. All information will be processed in accordance with GDPR (EU 2016/679). The Poznan University of Medical Sciences is the data controller for scientific purposes only.

Contact:
weronikapucek16@gmail.com
+48 881 084 656

Sincerely,
Weronika Pucek

## 1. Please indicate your gender *

Select one answer.

○ Female

○ Male

## 2. Please indicate your age *

________________________

## 3. Place of residence (Voivodeship) *

Select one answer.

○ Lower Silesian

○ Kuyavian–Pomeranian

○ Lublin

○ Lubusz

○ Łódź

○ Lesser Poland

○ Masovian

○ Opole

○ Subcarpathian

○ Podlaskie

○ Pomeranian

○ Silesian

○ Świętokrzyskie

○ Warmian–Masurian

○ Greater Poland

○ West Pomeranian

## 4. Years of professional experience as a physician *

________________________

## 5. In which hospital department(s) do you currently work? *

Select all that apply.

☐ Department of Anesthesiology and Intensive Care

☐ Department of General Surgery

☐ Department of Vascular Surgery

☐ Department of Trauma and Orthopedic Surgery

☐ Department of Internal Medicine

☐ Department of Cardiology

☐ Department of Diabetology

☐ Department of Endocrinology

☐ Department of Neurology

☐ Department of Neurosurgery

☐ Department of Nephrology

☐ Department of Obstetrics and Gynecology

☐ Department of Neonatology

☐ Department of Rheumatology

☐ Emergency Department (ED)

☐ Other

## 6. Approximately what percentage of your patients receive GLP-1 receptor agonists for weight reduction annually? *

Select one answer.

○ Less than 1%

○ 1–10%

○ 11–20%

○ 21–30%

○ 31–40%

○ 41–50%

○ More than 50%

## 7. Which criterion is your primary indication for prescribing GLP-1 receptor agonists? *

Select one answer.

○ Glycemic control

○ Weight reduction

○ Both of the above

## 8. In your opinion, does treatment with GLP-1 receptor agonists reduce the risk of metabolic syndrome? *

Select one answer.

○ Yes

○ No

○ I do not know

## 9. How would you rate the effectiveness of GLP-1 receptor agonists in reducing body weight compared with other available therapies? *

Select one answer.

○ Very effective

○ Effective

○ Ineffective

## 10. Which therapeutic recommendations do you provide to patients receiving GLP-1 receptor agonists in order to maximize weight loss? *

Select all that apply.

☐ Regular physical activity

☐ Individualized dietary plan

☐ Other

## 11. Do you consider GLP-1 receptor agonist therapy to be safe? *

Select one answer.

○ Yes

○ No

○ Difficult to determine

## 12. Have you observed a significant reduction in HbA1c levels in patients receiving GLP-1 receptor agonists? *

Select one answer.

○ Yes

○ No

○ Difficult to determine

## 13. Are patients receiving GLP-1 receptor agonists regularly monitored through follow-up examinations? *

Select one answer.

○ Yes

○ No

## 14. How frequently do you monitor the progress of patients receiving GLP-1 receptor agonists for weight reduction? *

Select one answer.

○ Regularly

○ Periodically

○ Rarely

## 15. Have you observed a significant reduction in LDL cholesterol levels in patients receiving GLP-1 receptor agonists? *

Select one answer.

○ Yes

○ No

○ Difficult to determine

## 16. Have you observed a significant reduction in HDL cholesterol levels in patients receiving GLP-1 receptor agonists? *

Select one answer.

○ Yes

○ No

○ Difficult to determine

## 17. Have you observed a significant reduction in non-HDL cholesterol levels in patients receiving GLP-1 receptor agonists? *

Select one answer.

○ Yes

○ No

○ Difficult to determine

## 18. Have you observed a significant reduction in triglyceride levels in patients receiving GLP-1 receptor agonists? *

Select one answer.

○ Yes

○ No

○ Difficult to determine

## 19. Have you observed a significant reduction in total cholesterol levels in patients receiving GLP-1 receptor agonists? *

Select one answer.

○ Yes

○ No

○ Difficult to determine

## 20. Do you believe that GLP-1 receptor agonists reduce anxiety when used in the treatment of obesity and/or type 2 diabetes? *

Select one answer.

○ Yes

○ No

○ I do not know

## 21. Do you believe that GLP-1 receptor agonists reduce stress when used in the treatment of obesity and/or type 2 diabetes? *

Select one answer.

○ Yes

○ No

○ I do not know

## 22. Do you believe that GLP-1 receptor agonists reduce symptoms of depression when used in the treatment of obesity and/or type 2 diabetes? *

Select one answer.

○ Yes

○ No

○ I do not know

## 23. Do you believe that GLP-1 receptor agonists reduce the number of cigarettes smoked by patients? *

Select one answer.

○ Yes

○ No

○ I do not know

## 24. Have you observed adverse events in patients receiving GLP-1 receptor agonist therapy? *

Select one answer.

○ Yes

○ No

## 25. If you answered "Yes" to the previous question, please indicate the adverse events observed.

Select all that apply.

☐ Gastrointestinal disorders (nausea, vomiting, diarrhea)

☐ Redness, swelling, or pain at the injection site

☐ Hypoglycemia

☐ Dizziness and/or weakness

☐ Cardiac arrhythmias

☐ Other

## 26. Do you believe that GLP-1 receptor agonists reduce alcohol dependence among patients? *

Select one answer.

○ Yes

○ No

○ I do not know

## 27. In your opinion, do adverse events occur more frequently during GLP-1 receptor agonist therapy than during treatment with other anti-obesity medications? *

Select one answer.

○ Yes

○ No

○ Difficult to determine

## 28. Have you observed clinically significant interactions between GLP-1 receptor agonists and other long-term medications used by your patients? *

Select one answer.

○ Yes

○ No

○ Difficult to determine

## 29. Do patients ask you to prescribe GLP-1 receptor agonists? *

Select one answer.

○ Yes

○ No

## 30. How would you assess physicians' level of knowledge regarding the use of GLP-1 receptor agonists in the treatment of metabolic disorders? *

Select one answer.

○ High

○ Moderate

○ Low

## 31. How would you assess patients' adherence to medical recommendations regarding GLP-1 receptor agonist pharmacotherapy for obesity? *

Select one answer.

○ High

○ Moderate

○ Low

## 32. In your opinion, do the high costs of GLP-1 receptor agonist therapy constitute a barrier to their widespread use in clinical practice? *

Select one answer.

○ Yes

○ No

○ Difficult to determine

## 33. Do you believe that GLP-1 receptor agonists should be reimbursed? *

Select one answer.

○ Yes

○ No

○ I do not know

## 34. Do you support the wider use of GLP-1 receptor agonists for the treatment of obesity? *

Select one answer.

○ Yes

○ No

## 35. In your opinion, do GLP-1 receptor agonists represent a breakthrough in the pharmacological treatment of obesity and/or type 2 diabetes? *

Select one answer.

○ Yes

○ No

○ I do not know

# Clinical and Social Aspects of the Use of the Latest Pharmacotherapy for Obesity and/or Type 2 Diabetes

Dear Sir or Madam,

The following questionnaire has been developed for use in the doctoral dissertation entitled: “Clinical and Social Aspects of the Use of the Latest Pharmacotherapy for Obesity and/or Type 2 Diabetes.”

Below you will find detailed information regarding the survey.

The aim of this study is to evaluate the practical application of the latest incretin hormone analogues in preventing the prevalence of selected cardiovascular risk factors (obesity, type 2 diabetes, depressive disorders, alcohol dependence, and nicotine dependence).

The study is being conducted to obtain the information necessary for the preparation of a doctoral dissertation.

If you are a pharmacist currently practicing in Poland, you are invited to participate in this study, which aims to determine whether the latest medications used in the treatment of obesity and/or type 2 diabetes offer hope for reducing the prevalence of selected cardiovascular risk factors (obesity, type 2 diabetes, depressive disorders, alcohol dependence, and nicotine dependence).

Completion of the questionnaire will take approximately 15–20 minutes. Please answer all questions to the best of your ability.

Participation is entirely anonymous and voluntary.

If you have any questions regarding the survey, please contact: weronikapucek16@gmail.com or +48 881084656.

Participation in this study does not infringe upon any of your rights.

All information collected will be processed and stored in accordance with the General Data Protection Regulation (GDPR 2016).

By participating in the study, you consent to the Karol Marcinkowski University of Medical Sciences in Poznań acting as the data controller for the information collected through this survey exclusively for scientific purposes.

You are cordially invited to participate in this survey.

Thank you very much for your commitment and support.
Yours sincerely,
Weronika Pucek

* Indicates a required question

1. Please indicate your gender: *

- Female
- Male

2. Please state your age: *

3. Place of residence (Voivodeship): *

- Lower Silesian
- Kuyavian-Pomeranian
- Lublin
- Lubusz
- Łódź
- Lesser Poland
- Masovian
- Opole
- Subcarpathian
- Podlaskie
- Pomeranian
- Silesian
- Świętokrzyskie
- Warmian-Masurian
- Greater Poland
- West Pomeranian

4. Years of professional experience as a pharmacist: *

5. Employment sector: *

- Community pharmacy
- Hospital pharmacy
- Pharmaceutical company
- Self-employed
- Other:

6. How often do patients present prescriptions for GLP-1 receptor agonists? *

- Daily
- Several times a week
- Occasionally
- Very rarely

7. In your opinion, is GLP-1 receptor agonist therapy frequently recommended by physicians for the treatment of obesity? *

- Yes
- No
- Difficult to determine

8. Do you believe pharmacists possess sufficient knowledge of obesity pharmacotherapy using GLP-1 receptor agonists to provide appropriate pharmaceutical care? *

- Yes
- No

9. Do you believe pharmacists/pharmacy technicians should further expand their knowledge regarding the use of GLP-1 receptor agonists in daily professional practice? *

- Yes
- No
- Difficult to determine

10. Based on patient feedback, how would you assess the effectiveness of GLP-1 receptor agonist therapy in the treatment of obesity? *

- Highly effective
- Effective
- Ineffective

11. How would you assess patients' level of awareness regarding GLP-1 receptor agonist therapy for obesity? *

- High
- Moderate
- Low

12. Do you consider GLP-1 receptor agonist therapy to be safe? *

- Yes
- No
- Difficult to determine

13. Do patients report adverse effects during GLP-1 receptor agonist therapy? *

- Yes
- No

14. If yes, please indicate the reported adverse effects (select all that apply):

- Gastrointestinal disorders (nausea, vomiting, diarrhea)
- Redness/swelling/pain at the injection site
- Hypoglycemia
- Dizziness/weakness
- Cardiac arrhythmias
- Other:

15. Do you believe adverse effects occur more frequently during GLP-1 receptor agonist therapy than with other anti-obesity pharmacotherapies? *

- Yes
- No
- Difficult to determine

16. Have you observed any problems with the availability of GLP-1 receptor agonists in pharmacies or pharmaceutical wholesalers? *

- Yes
- No
- Difficult to determine

17. In your opinion, do the high costs of GLP-1 receptor agonists constitute a barrier for patients? *

- Yes
- No
- Difficult to determine

18. Do you believe GLP-1 receptor agonists should be reimbursed? *

- Yes
- No
- I do not know

19. Do you support the wider use of GLP-1 receptor agonists for the treatment of obesity? *

- Yes
- No

20. In your opinion, do GLP-1 receptor agonists represent a breakthrough in the pharmacotherapy of obesity and/or type 2 diabetes? *

- Yes
- No
- I do not know

# Clinical and Social Aspects of the Use of the Latest Pharmacotherapy for Obesity and/or Type 2 Diabetes

Dear Sir/Madam,

This questionnaire was developed for use in the doctoral dissertation entitled “Clinical and Social Aspects of the Use of the Latest Pharmacotherapy for Obesity and/or Type 2 Diabetes.”

The aim of this study is to assess the practical use of the latest incretin hormone analogues in preventing selected cardiovascular risk factors (obesity, type 2 diabetes, depressive disorders, alcohol dependence, and nicotine dependence).

The study is conducted to obtain data required for the preparation of a doctoral dissertation.

If you are a registered nurse currently practicing in Poland, you are invited to participate. The survey seeks to determine whether the latest medications used for obesity and/or type 2 diabetes may help reduce the prevalence of selected cardiovascular risk factors.

Completion of the questionnaire takes approximately 25–30 minutes. Participation is voluntary and anonymous.

Questions: weronikapucek16@gmail.com | +48 881 084 656

All collected data will be processed in accordance with the General Data Protection Regulation (GDPR 2016/679). By participating, you consent to the Poznan University of Medical Sciences acting as the data controller solely for scientific purposes.

Sincerely,
Weronika Pucek

## 1. Please indicate your gender *

- ☐ Female
- ☐ Male

## 2. Please indicate your age *

## 3. Place of residence (Voivodeship/Province) *

- ☐ Lower Silesian
- ☐ Kuyavian-Pomeranian
- ☐ Lublin
- ☐ Lubusz
- ☐ Łódź
- ☐ Lesser Poland
- ☐ Masovian
- ☐ Opole
- ☐ Subcarpathian
- ☐ Podlaskie
- ☐ Pomeranian
- ☐ Silesian
- ☐ Świętokrzyskie
- ☐ Warmian-Masurian
- ☐ Greater Poland
- ☐ West Pomeranian

## 4. Years of professional experience as a nurse *

## 5. Hospital department(s) where you work (select all that apply) *

- ☐ Department of Anesthesiology and Intensive Care
- ☐ General Surgery
- ☐ Vascular Surgery
- ☐ Trauma and Orthopedic Surgery
- ☐ Internal Medicine
- ☐ Cardiology
- ☐ Diabetology
- ☐ Endocrinology
- ☐ Neurology
- ☐ Neurosurgery
- ☐ Nephrology
- ☐ Obstetrics and Gynecology
- ☐ Neonatology
- ☐ Rheumatology
- ☐ Emergency Department (ED)
- ☐ Other

## 6. Approximately what percentage of your patients receive GLP-1 receptor agonists for weight reduction each year? *

- ☐ Less than 1%
- ☐ 1–10%
- ☐ 11–20%
- ☐ 21–30%
- ☐ 31–40%
- ☐ 41–50%
- ☐ More than 50%

## 7. Have you observed adverse effects in patients receiving GLP-1 receptor agonist therapy? *

- ☐ Yes
- ☐ No

## 8. If yes, which adverse effects have you observed? (Select all that apply)

- ☐ Gastrointestinal symptoms (nausea, vomiting, diarrhea)
- ☐ Injection-site redness/swelling/pain
- ☐ Hypoglycemia
- ☐ Dizziness/weakness
- ☐ Cardiac arrhythmias
- ☐ Other

## 9. Do you consider GLP-1 receptor agonist therapy to be safe? *

- ☐ Yes
- ☐ No
- ☐ Difficult to determine

## 10. Do you believe adverse effects occur more frequently with GLP-1 receptor agonists than with other anti-obesity medications? *

- ☐ Yes
- ☐ No
- ☐ Difficult to determine

## 11. How would you rate the effectiveness of GLP-1 receptor agonists for weight reduction compared with other available therapies? *

- ☐ Very effective
- ☐ Effective
- ☐ Ineffective

## 12. Have you observed a significant reduction in HbA1c levels among patients receiving GLP-1 receptor agonists? *

- ☐ Yes
- ☐ No
- ☐ Difficult to determine

## 13. Have you observed a significant reduction in LDL cholesterol levels among patients receiving GLP-1 receptor agonists? *

- ☐ Yes
- ☐ No
- ☐ Difficult to determine

## 14. Have you observed a significant reduction in HDL cholesterol levels among patients receiving GLP-1 receptor agonists? *

- ☐ Yes
- ☐ No
- ☐ Difficult to determine

## 15. Have you observed a significant reduction in non-HDL cholesterol levels among patients receiving GLP-1 receptor agonists? *

- ☐ Yes
- ☐ No
- ☐ Difficult to determine

## 16. Have you observed a significant reduction in triglyceride levels among patients receiving GLP-1 receptor agonists? *

- ☐ Yes
- ☐ No
- ☐ Difficult to determine

## 17. Have you observed a significant reduction in total cholesterol levels among patients receiving GLP-1 receptor agonists? *

- ☐ Yes
- ☐ No
- ☐ Difficult to determine

## 18. Do you believe GLP-1 receptor agonists reduce the number of cigarettes smoked by patients? *

- ☐ Yes
- ☐ No
- ☐ Difficult to determine

## 19. Do you believe GLP-1 receptor agonists reduce alcohol dependence among patients? *

- ☐ Yes
- ☐ No
- ☐ Difficult to determine

## 20. How would you assess nursing staff knowledge regarding the use of GLP-1 receptor agonists in treating metabolic disorders? *

- ☐ High
- ☐ Moderate
- ☐ Low

## 21. How would you assess patients' adherence to medical recommendations regarding GLP-1 receptor agonist therapy for obesity? *

- ☐ High
- ☐ Moderate
- ☐ Low

## 22. Do you believe GLP-1 receptor agonists reduce anxiety when used to treat obesity and/or type 2 diabetes? *

- ☐ Yes
- ☐ No
- ☐ Difficult to determine

## 23. Do you believe GLP-1 receptor agonists reduce stress when used to treat obesity and/or type 2 diabetes? *

- ☐ Yes
- ☐ No
- ☐ Difficult to determine

## 24. Do you believe GLP-1 receptor agonists reduce symptoms of depression when used to treat obesity and/or type 2 diabetes? *

- ☐ Yes
- ☐ No
- ☐ Difficult to determine

## 25. In your opinion, do the high costs of GLP-1 receptor agonist therapy constitute a barrier to their widespread use? *

- ☐ Yes
- ☐ No
- ☐ I don't know

## 26. Do you believe GLP-1 receptor agonists should be reimbursed? *

- ☐ Yes
- ☐ No
- ☐ I don't know

## 27. In your opinion, do GLP-1 receptor agonists represent a breakthrough in the pharmacotherapy of obesity and/or type 2 diabetes? *

- ☐ Yes
- ☐ No
- ☐ I don't know

# Clinical and Social Aspects of the Use of the Latest Pharmacotherapy for Obesity and/or Type 2 Diabetes

## Information for Participants

Dear Sir/Madam,

This questionnaire has been developed for use in the doctoral dissertation entitled “Clinical and Social Aspects of the Use of the Latest Pharmacotherapy for Obesity and/or Type 2 Diabetes”.

The aim of this study is to evaluate the practical application of the latest incretin hormone analogues in preventing selected cardiovascular risk factors, including obesity, type 2 diabetes mellitus, depressive disorders, alcohol dependence, and nicotine dependence.

If you are a dietitian currently practicing in Poland, you are invited to participate in this anonymous and voluntary study. Completion of the questionnaire will take approximately 20–25 minutes.

All information will be processed in accordance with the General Data Protection Regulation (GDPR, EU 2016/679). The Poznan University of Medical Sciences is the data controller solely for scientific purposes.

Contact:
weronikapucek16@gmail.com
+48 881 084 656

Sincerely,
Weronika Pucek

## 1. Please indicate your gender *

Select one answer.

○ Female

○ Male

## 2. Please indicate your age *

________________

## 3. Place of residence (Voivodeship) *

Select one answer.

○ Lower Silesian

○ Kuyavian–Pomeranian

○ Lublin

○ Lubusz

○ Łódź

○ Lesser Poland

○ Masovian

○ Opole

○ Subcarpathian

○ Podlaskie

○ Pomeranian

○ Silesian

○ Świętokrzyskie

○ Warmian–Masurian

○ Greater Poland

○ West Pomeranian

## 4. Years of professional experience as a dietitian *

________________

## 5. Place(s) of employment *

Select all that apply.

☐ Hospital

☐ Outpatient clinic

☐ Dietetic office

☐ Private practice

☐ Blogger/Influencer

☐ Kindergarten/Nursery

☐ Diet catering company

☐ Other

## 6. Approximately what percentage of your patients receive GLP-1 receptor agonists for weight reduction annually? *

Select one answer.

○ Less than 1%

○ 1–10%

○ 11–20%

○ 21–30%

○ 31–40%

○ 41–50%

○ More than 50%

## 7. How would you rate the effectiveness of GLP-1 receptor agonists in reducing body weight compared with other available therapies? *

Select one answer.

○ Very effective

○ Effective

○ Ineffective

## 8. In your opinion, does treatment with GLP-1 receptor agonists reduce the risk of metabolic syndrome? *

Select one answer.

○ Yes

○ No

○ I do not know

## 9. How would you assess patients' adherence to medical recommendations regarding GLP-1 receptor agonist therapy for obesity? *

Select one answer.

○ High

○ Moderate

○ Low

## 10. Do you consider GLP-1 receptor agonist therapy to be safe? *

Select one answer.

○ Yes

○ No

○ Difficult to determine

## 11. Do you believe GLP-1 receptor agonists reduce anxiety when used in the treatment of obesity and/or type 2 diabetes? *

Select one answer.

○ Yes

○ No

○ I do not know

## 12. Do you believe GLP-1 receptor agonists reduce stress when used in the treatment of obesity and/or type 2 diabetes? *

Select one answer.

○ Yes

○ No

○ I do not know

## 13. Do you believe GLP-1 receptor agonists reduce symptoms of depression when used in the treatment of obesity and/or type 2 diabetes? *

Select one answer.

○ Yes

○ No

○ I do not know

## 14. Do you believe GLP-1 receptor agonists reduce the number of cigarettes smoked by patients? *

Select one answer.

○ Yes

○ No

○ I do not know

## 15. Do you believe GLP-1 receptor agonists reduce alcohol dependence among patients? *

Select one answer.

○ Yes

○ No

○ I do not know

## 16. Have you observed significant improvements in glycemic control among patients receiving GLP-1 receptor agonists? *

Select one answer.

○ Yes, glycemic control improved significantly

○ No change in glycemic control

○ No clinically significant improvement

## 17. Have you observed adverse events in patients receiving GLP-1 receptor agonist therapy? *

Select one answer.

○ Yes

○ No

## 18. If yes, which adverse events have you observed?

Select all that apply.

☐ Gastrointestinal disorders (nausea, vomiting, diarrhea)

☐ Redness/swelling/pain at the injection site

☐ Hypoglycemia

☐ Dizziness/weakness

☐ Cardiac arrhythmias

☐ Other

## 19. Do you believe GLP-1 receptor agonists increase the risk of gallstone disease? *

Select one answer.

○ Yes

○ No

○ I do not know

## 20. Do you believe the high cost of GLP-1 receptor agonist therapy is a barrier to their widespread use? *

Select one answer.

○ Yes

○ No

○ I do not know

## 21. Do you believe GLP-1 receptor agonists should be reimbursed? *

Select one answer.

○ Yes

○ No

○ I do not know

## 22. In your opinion, do GLP-1 receptor agonists represent a breakthrough in the pharmacotherapy of obesity and/or type 2 diabetes? *

Select one answer.

○ Yes

○ No

○ I do not know

# Clinical and Social Aspects of the Use of the Latest Pharmacotherapy for Obesity and/or Type 2 Diabetes

## Information for Participants

Dear Sir/Madam,

This questionnaire has been developed for use in the doctoral dissertation entitled
“Clinical and Social Aspects of the Use of the Latest Pharmacotherapy for Obesity and/or Type 2 Diabetes”.

The aim of this study is to evaluate the practical application of the latest incretin hormone analogues in preventing selected cardiovascular risk factors, including obesity, type 2 diabetes mellitus, depressive disorders, alcohol dependence, and nicotine dependence.

If you are a physiotherapist currently practicing in Poland, you are invited to participate in this anonymous and voluntary study. Completion of the questionnaire takes approximately 15–20 minutes.

All data will be processed in accordance with the General Data Protection Regulation (GDPR, EU 2016/679).

Contact:
weronikapucek16@gmail.com
+48 881 084 656

Sincerely,
Weronika Pucek

## 1. Please indicate your gender *

Select one answer.

○ Female

○ Male

## 2. Please indicate your age *

______________

## 3. Place of residence (Voivodeship) *

Select one answer.

○ Lower Silesian

○ Kuyavian–Pomeranian

○ Lublin

○ Lubusz

○ Łódź

○ Lesser Poland

○ Masovian

○ Opole

○ Subcarpathian

○ Podlaskie

○ Pomeranian

○ Silesian

○ Świętokrzyskie

○ Warmian–Masurian

○ Greater Poland

○ West Pomeranian

## 4. Years of professional experience as a physiotherapist *

______________

## 5. Place(s) of employment *

Select all that apply.

☐ Hospital

☐ Outpatient clinic

☐ Rehabilitation center

☐ Nursing home

☐ Sanatorium

☐ Sports club

☐ Fitness center

☐ Other

## 6. Approximately what percentage of your patients receive GLP-1 receptor agonists for weight reduction annually? *

Select one answer.

○ Less than 1%

○ 1–10%

○ 11–20%

○ 21–30%

○ 31–40%

○ 41–50%

○ More than 50%

## 7. How would you rate the effectiveness of GLP-1 receptor agonists for weight reduction compared with other available therapies? *

Select one answer.

○ Very effective

○ Effective

○ Ineffective

## 8. In your opinion, does treatment with GLP-1 receptor agonists reduce the risk of metabolic syndrome? *

Select one answer.

○ Yes

○ No

○ I do not know

## 9. Do you consider GLP-1 receptor agonist therapy to be safe? *

Select one answer.

○ Yes

○ No

○ Difficult to determine

## 10. Do you believe treatment with GLP-1 receptor agonists may cause inflammation of muscles and joints? *

Select one answer.

○ Yes

○ No

○ I do not know

## 11. Do you believe GLP-1 receptor agonists reduce anxiety when used in the treatment of obesity and/or type 2 diabetes? *

Select one answer.

○ Yes

○ No

○ I do not know

## 12. Do you believe GLP-1 receptor agonists reduce stress when used in the treatment of obesity and/or type 2 diabetes? *

Select one answer.

○ Yes

○ No

○ I do not know

## 13. Do you believe GLP-1 receptor agonists reduce symptoms of depression when used in the treatment of obesity and/or type 2 diabetes? *

Select one answer.

○ Yes

○ No

○ I do not know

## 14. Do you believe the high cost of GLP-1 receptor agonist therapy is a barrier to their widespread use? *

Select one answer.

○ Yes

○ No

○ I do not know

## 15. Do you believe GLP-1 receptor agonists should be reimbursed? *

Select one answer.

○ Yes

○ No

○ I do not know

## 16. In your opinion, do GLP-1 receptor agonists represent a breakthrough in the pharmacotherapy of obesity and/or type 2 diabetes? *

Select one answer.

○ Yes

○ No

○ I do not know
